# Capsule-dependent impact of MAPK signalling on host cell invasion and immune response during infection of the choroid plexus epithelium by Neisseria meningitidis

**DOI:** 10.1186/s12987-021-00288-7

**Published:** 2021-12-04

**Authors:** Rosanna Herold, René Scholtysik, Selina Moroniak, Christel Weiss, Hiroshi Ishikawa, Horst Schroten, Christian Schwerk

**Affiliations:** 1 Pediatric Infectious Diseases, Department of Pediatrics, Medical Faculty Mannheim, Heidelberg University, Theodor-Kutzer-Ufer 1-3, 68167 Mannheim, Germany; 2 Genomics & Transcriptomics Facility, Institute of Cell Biology, University Hospital Essen, Virchowstraße 173, 45122 Essen, Germany; 3 Department of Medical Statistics and Biomathematics, Medical Faculty Mannheim, Heidelberg University, Theodor-Kutzer-Ufer 1-3, 68167 Mannheim, Germany; 4 Laboratory of Clinical Regenerative Medicine, Department of Neurosurgery, Faculty of Medicine, University of Tsukuba, 1-1-1Tennodai, Tsukuba, Ibaraki 305-8575 Japan

**Keywords:** Blood-cerebrospinal fluid barrier, Host innate signalling, Host pathogen interaction, MAPK, Meningitis, *Neisseria meningitidis*

## Abstract

**Background:**

The Gram-negative bacterium *Neisseria meningitidis* (*Nm*) can cause meningitis in humans, but the host signalling pathways manipulated by *Nm* during central nervous system (CNS) entry are not completely understood.

**Methods:**

We investigate the role of the mitogen-activated protein kinases (MAPK) Erk1/2 and p38 in an in vitro model of the blood-cerebrospinal fluid barrier (BCSFB) based on human epithelial choroid plexus (CP) papilloma (HIBCPP) cells during infection with *Nm* serogroup B (*Nm*B) and serogroup C (*Nm*C) strains. A transcriptome analysis of HIBCPP cells following infection with *Nm* by massive analysis of cDNA ends (MACE) was done to further characterize the cellular response to infection of the barrier.

**Results:**

Interestingly, whereas *Nm*B and *Nm*C wild type strains required active Erk1/2 and p38 pathways for infection, invasion by capsule-deficient mutants was independent of Erk1/2 and, in case of the *Nm*B strain, of p38 activity. The transcriptome analysis of HIBCPP cells following infection with *Nm* demonstrated specific regulation of genes involved in the immune response dependent on Erk1/2 signalling. Gene ontology (GO) analysis confirmed loss of MAPK signalling after Erk1/2 inhibition and revealed an additional reduction of cellular responses including NFκB and JAK-STAT signalling. Interestingly, GO terms related to TNF signalling and production of IL6 were lost specifically following Erk1/2 inhibition during infection with wild type *Nm*, which correlated with the reduced infection rates by the wild type in absence of Erk1/2 signalling.

**Conclusion:**

Our data point towards a role of MAPK signalling during infection of the CP epithelium by *Nm*, which is strongly influenced by capsule expression, and affects infection rates as well as the host cell response.

**Supplementary Information:**

The online version contains supplementary material available at 10.1186/s12987-021-00288-7.

## Background

*Neisseria meningitidis (Nm)* is a human-specific, Gram-negative diplococcus that extracellularly colonizes the nasopharynx and is asymptomatic in 10% of the population [1]. Although *Nm* is often found to be non-pathogenic and commensal, it can also cause severe invasive infection, such as acute meningitis and sepsis. Crossing of the mucosal barrier is prerequisite for meningococcal pathogenicity and followed by dissemination in the bloodstream [2]. To cause meningitis, *Nm* needs to enter the central nervous system (CNS), which is well protected from pathogens due to the presence of protective barriers, such as the blood–brain barrier (BBB) and the blood-cerebrospinal fluid barrier (BCSFB). *Nm* has been proposed to cross these protective barriers of the brain, the BBB and the BCSFB, to reach the inner and outer cerebrospinal fluid (CSF) [3–7]. There is evidence that, during an infection, *Nm* can interact with the BCSFB, whose morphological correlate are the epithelial cells of the choroid plexus (CP) [8]. In an in vitro model of the BCSFB, which is based on human choroid plexus papilloma (HIBCPP) cells, *Nm* have been shown to interact, enter and transmigrate across the epithelial cell layer following infection from the physiologically relevant basolateral side [3, 4].

To enter the CNS, *Nm* use a variety of virulence factors involved in the different steps of pathogenesis. The polysaccharide capsule has been described as the main virulence factor of *Nm* and is a major contributor to meningococcal disease. It is essential for bloodstream survival and masks the function of non-pilus adhesins [9]. The capsule of Neisseria strains associated with invasive diseases has been shown to contain sialic acid, with the *Nm* serogroup B (*Nm*B) owning a α2-8-linked N-acetylneuraminic acid, and the *Nm* serogroup C (*Nm*C) presenting α 2–9-linked partially O-acetylneuraminic acid [10]. However, adhesion and invasion of host tissues is attenuated by the capsule as was shown for the capsulated *Nm* serogroup B strain (*NmB*) MC58 during infection of HIBCPP cells. In this model system, the capsule-deficient mutant (MC58siaD^−^) on the other hand, displayed higher invasion rates than the wild type strain [3, 4]. Changes in capsule expression, such as hypo- or hyperencapsulation, capsule “switching” and acetylation, can lead to immune escape and the loss or downregulation of the capsule has been shown to not only facilitate attachment to host cells, but also the formation of microcolonies and the carriage state at human mucosal surfaces [11].

To facilitate infection of host cells, meningitis-causing pathogens can use host cell signalling molecules and manipulate intracellular host cell signalling cascades to their advantage. One of those signalling cascades is the mitogen-activated protein kinase (MAPK) pathway [12]. Among the best described members of the MAPK family, which are activated by phosphorylation, are the extracellular signal-regulated kinases (Erk) 1 and 2, members of the p38 kinases and the JUN N-terminal kinases (JNK) [13]. For *Listeria monocytogenes* (*Lm*), invasion in HIBCPP cells was shown to be dependent on the activation of Erk1/2 and p38 [14]. The activation of MAPKs p38 and JNK has been described in vitro for *Nm* in endothelial cells of the BBB resulting in the release of Interleukin (IL)6 and IL8 [15].

CNS entry of pathogens can cause an immune response by the host in the process [16]. An elevation of cytokine levels in the CSF of patients suffering from bacterial meningitis has been described in several studies. For the pathogenesis of bacterial meningitis, the cytokines and chemokines that are characteristically found in the CSF of patients, include IL6, IL8, TNFα, CXCL1 IL1β and MCP-1 [17–19]. Furthermore, the CP has been proposed to contribute to the production of cytokines in the CNS during inflammation and following infection with pathogens [20–22].

In this study, we examined the role of MAPK during infection of the BCSFB by *Nm* as well as the cellular answer of HIBCPP cells to basolateral infection in vitro. Emphasis was put on the role of the capsule during infection of *Nm* strains of the serogroups B and C. We show that both Erk1/2 and p38 are required for infection of the HIBCPP cells by the *Nm* wild type strains. In contrast, inhibition of MAPK activity revealed a variable impact on infection by capsule-depleted strains. Interestingly, Erk1/2 inhibition caused an increased infection by the capsule-deficient *Nm*B mutant. Subsequently, we determined the transcriptome of HIBCPP cells after infection with the *Nm*B strains with a special focus on the Erk1/2 signalling cascade during infection. The induction of an inflammatory response following infection was regulated in a capsule and Erk1/2 dependent manner. Loss of GO terms related to TNF signalling and production of IL6 following Erk1/2 inhibition during infection with wild type *Nm* correlated with the reduced infection of HIBCPP cells by the wild type in absence of Erk1/2 signalling. Our data underline capsule-dependent infection mechanisms of *Nm* in HIBCPP cells as well as a central role of Erk1/2 signalling during infection and point towards a fine-tuned inflammatory response influenced by capsule expression and Erk1/2 signalling.

## Materials and methods

### Bacterial strains and growth conditions

The bacterial strains used in this study are listed in Table 1. Stock cultures were stored at − 80 °C in DMEM/F12 supplemented with 1% fetal calf serum (FCS), 5 mg mL^−1^ insulin and 15% glycerol. For infection experiments, the stocks were plated on Chocolate Agar with Vitox (Oxoid, Wesel, Germany) and grown at 37 °C in 5% CO_2_ atmosphere overnight. Single colonies of the overnight culture were picked and were dissolved and washed in phenol-red free DMEM/F12 with 1% FCS and 5 mg mL^−1^ insulin and adjusted to an optical density of 0.1 at 600 nm (OD_600_) which corresponded to approximately 1 × 10^8^ colony forming units (CFU) per mL.﻿ During the infection experiments, a multiplicity of infection (MOI) of 10 or 100 was used as described for the individual experiments. Bacterial growth was monitored throughout all experiments and in the presence and absence of inhibitors.

**Table 1:** Bacterial strains

N. meningitidis	Serogroup		References
MC58	B	Wild type	[23]
MC58siaD^−^	Capsule-deficient mutant	Isogenic siaD mutant	[24]
WUE2120	C	Wild type	[25]
WUE2120siaD^−^	Capsule-deficient mutant	Isogenic siaD mutant	[24]

### Cultivation of HIBCPP cells on cell culture filter inserts and surveillance of barrier function

The HIBCPP cells were cultured in DMEM/F12 with 10% FCS and 5 mg mL^−1^ insulin. Prior to the infection experiments, the cells were grown in the inverted cell culture system as previously described [3]. In brief, the cells were seeded at a density of 8 × 10^4^ cells on uncoated filter inserts with a PET membrane. The confluency of the cell layer and barrier integrity of the cells were determined by measuring the transepithelial electrical resistance (TEER) using an epithelial voltohmmeter, the Millicell-ERS STX-2 electrode system (Millipore, Schwalbach, Germany), and by measuring the paracellular permeability of the HIBCPP cells on the filter inserts by adding a FITC-inulin tracer solution (100 mg mL^−1^; Sigma, Deisenhofen, Germany) to the filter compartment of the inserts. Passage of the FITC-Inulin to the lower compartment over the course of the experiment was measured using a Tecan Infinite M200 Multiwell reader (Tecan, Switzerland) as previously described [26]. The confluent cell layer has approximately 4 × 10^5^ cells per filter insert.

### Infection of HIBCPP cells with *Nm* and determination of bacterial invasion by double immunofluorescence

Infection of the HIBCPP cells with *Nm* and subsequent determination of the rate of infection was previously described [4]. Minor modifications are described in the following. HIBCPP cells were grown in the inverted culture system until confluency and infected with the *Nm* strains at a MOI of 10 for 4 h (quantification of intracellular bacteria and Western blot) or 6 h (isolation of RNA for transcriptome analysis and qPCR), respectively, in DMEM/F12 with 1% FCS and 5 mg mL^−1^ insulin. Infection experiments were performed in the presence and absence of the inhibitors or stimuli as described in detail for the specific experiments. After the infection, the extracellular *Nm* were detected using the primary antibody (1:1000) anti-Meningococcal Serosubtype P1.7 (NIBSC, Blanche Lane, Ridge, Hertfordshire, UK). After formaldehyde fixation (3.7%), the secondary antibody (Alexa Fluor® 594 donkey anti-Mouse; 1:500; Life Technologies) was added for visualization. Permeabilization with PBS/0.5% Triton X-100/1% bovine serum albumin was used to allow detection of intracellular *Nm* additionally to extracellularly adhered bacteria with antibody P1.7 (1:1000), followed by staining with the secondary antibody (Alexa Fluor® 488 donkey anti-Mouse; 1:500; Life Technologies), along with addition of 4′,6-diamidino-2-phenylindole dihydrochloride (DAPI; Calbiochem, Darmstadt, Germany) (1:50,000) for visualization of nuclei and staining of the actin cytoskeleton with Alexa Fluor 660 phalloidin (1:250; Life Technologies). Quantification of the intracellular *Nm* was performed as previously described [4]. For inhibition experiments, cells were pre-treated for 1 h with the inhibitors U0126 and SB203580 (Merck Millipore, Darmstadt, Germany).

### Immunoblot

Bacterial infection of the HIBCPP cells was followed by a wash step with PBS and the subsequent extraction of whole protein lysate using modified RIPA buffer (1 × RIPA lysis buffer, 50 mM NaF, 1 mM Na_3_VO_4_, 1 mM PMSF, protease inhibitor cocktail). Whole protein content was determined using the DC Protein Assay (BioRad, München, Germany) according to manufacturer´s instructions. 20 µg of protein was resolved by 4–12% Bis–Tris gels (Invitrogen, Karlsruhe, Germany) and subsequently electrotransferred onto nitrocellulose membranes. Target proteins were detected using the antibodies: rabbit anti-phospho-Erk1/2, rabbit anti-rk1/2, rabbit anti-phospho-p38, rabbit anti-p38, rabbit anti-phospho-MAPKAPK-2 and rabbit anti-MAPKAPK-2 which were obtained from Cell Signalling (Cambridge, UK). To visualize immunoreactivity, the Immobilon Western Kit (Millipore, Schwalbach, Germany) was used. All blots were performed at least three times, each representing an independent experiment.

### RNA extraction and quality control

The infection of the HIBCPP cells with the *Nm* strains was followed by a wash-step with PBS to remove unbound bacteria. The total RNA of the HIBCPP cells was extracted using modified RLT buffer (1 × RLT, 10 mM β-Mercaptoethanol) obtained from Qiagen, Hilden, Germany followed by the isolation of RNA using the RNeasy® Mini Kit (Qiagen, Hilden, Germany) according to the manufacturer's instructions. Additionally, a DNA digestion using RNAse free DNAse (Qiagen, Hilden, Germany) was performed on the column. RNA quantity and purity were determined using spectrophotometry (ND1000, Peqlab Biotechnology, Erlangen, Germany). For QPCR analysis, 500 ng of total RNA were reverse transcribed using the AffinityScript QPCR cDNA Synthesis kit (Agilent Technologies, Santa Clara, CA, USA) following the manufacturer's instructions. The RNA quality of the samples used for the MACE analysis was additionally assessed using the Agilent 2100 Bioanalyzer System (Agilent Technologies, Waldbronn, Germany), in combination with the Agilent Bioanalyzer RNA Nano Chip to determine their RNA integrity number. Samples with RNA integrity numbers above 9.8 were used for the MACE analysis.

### Quantitative real-time PCR analysis (QPCR)

The expression of selected genes was quantitatively evaluated using the Brilliant 280 II SYBR® Green QPCR Master Mix kit (Agilent Technologies) according to the manufacturer’s instructions. The qPCR was run using the Stratagene Mx3005P system with the MX software. The set-up of PCR conditions was as follows: initial denaturation (99 °C, 10 min) and subsequently 40 cycles of denaturation (95 °C, 30 s), annealing (60 °C, 1 min), extension (72 °C, 1 min) using the 1 plateau pre-melt / RT segment and normal 2-step amplification setting, followed by determination of a dissociation curve (95 °C, 60 s; 65 °C, 30 s; 95 °C, 30 s). For the calculation of fold-changes, the 2^−ΔΔCt^ method [27] was applied using the gene for Glyceraldehyde 3-phosphate dehydrogenase (GAPDH) as control.

### RNA-seq analysis and bioinformatic processing post-sequencing

To analyse transcriptomic changes in HIBCPP cells after infection with *Nm*B strains, as well as the effect of the inhibition of Erk1/2 signalling during infection, MACE-seq was used. The libraries were prepared with the MACE-Kit for 3’-mRNA sequencing (GenXpro, Germany). The sequencing was performed on a Nextseq 550 (Illumina, USA) with a High Output Flow Cell, multiplexing all samples with 75 bp reads.

After demultiplexing with Casava (Illumina, USA), unique molecular barcodes (UMI) were extracted from each read. The reads were aligned to hg38 with bwa [28] and reads that had the same position and UMI were collapsed with picard tools [29]. The resulting consensus sequences were loaded into CLC Genomics Workbench (Qiagen, Germany), where the tool “RNA-seq Analysis” was applied to each sample. The data generated during RNA-seq was deposited in the Sequence Read Archive (SRA) on the National Center for Biotechnology Information (NCBI) platform. The BioProject accession number is PRJNA743158.

### RNA-seq statistical data analysis: differentially expressed genes (DEG) and gene ontology (GO)

The resulting expression data was then used for differential expression analysis with the respective tools in CLC for the different experimental groups. Infected cells were compared to the uninfected control while HIBCPP cells infected with *Nm*B in addition to the treatment with the inhibitor U0126 were compared to the control treated with the inhibitor. Lists of DEG were created by manually filtering in Excel from Microsoft Office. The Lists were filtered using the corrected False Discovery Rate (FDR) p-value ≤ 0.05 and log_2_ fold-changes of > 2. LAGO (Logically Accelerated GO Term Finder) was used to analyse GO. Overrepresented biological pathways were identified using a p-value cut-off of 0.01 and applying a Bonferroni correction [30, 31].

### Statistical analysis

The SAS system release 9.4 was applied for statistical analysis (SAS Institute Inc., Cary, NC, USA). For normally distributed data a one-way analysis of variance (ANOVA) was performed to compare the mean values of the differently treated cells. An adjustment for multiple comparisons was done by Scheffe`s test with the untreated, infected cells functioning as reference groups. P-values < 0.05 were considered to be statistically significant.

## Results

### Activation of MAPK Erk1/2 and p38 by *N. meningitides* in HIBCPP cells from the physiologically relevant basolateral side

We previously demonstrated that the *Nm* serogroups B and C invade preferentially from the physiologically relevant basolateral side [3, 4]. We were now interested in the activation of the MAPKs Erk1/2 and p38 during invasion of *Nm* from the basolateral side. For this purpose, the HIBCPP cells were grown in the inverted cell culture filter insert system and infected with bacteria for 4 h with a MOI of 100. Due to the filter membrane, the available HIBCPP cell surface area for direct contact with *Nm* is limited to about 10% in these experiments.

**Fig. 1:**
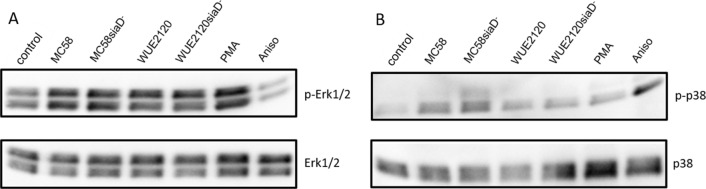
*Nm*B and *Nm*C strains activate p38 strongly, while Erk1/2 is only weakly activated. *Nm*B and *Nm*C strains as well as their capsule-deficient mutants activate the MAPK p38 when the basolateral side of the HIBCPP cells is infected, while the MAPK Erk1/2 is only weakly activated. The activation of the MAPK Erk1/2 (**A**) and p38 (**B**) was detected by Western blotting. HIBCPP cells were infected for 4 h with *Nm*B and *Nm*C wild type and capsule-deficient mutants in the inverted culture model of the HIBCPP cells from the basolateral side with a MOI of 100. Untreated cells (control) served as negative controls, cells treated with PMA or Anisomycin (Aniso) acted as positive controls for the activation of the Erk1/2 or p38 signal pathways, respectively. The blots were cropped to focus on the desired bands

The representative experiment shown in Fig. 1 demonstrates that infection of HIBCPP cells with both *NmB* and *NmC* strains results in a moderate activation of Erk1/2 and p38. In case of Erk1/2 phosphorylation, no major differences between the serogroups as well as presence and absence of the capsule could be observed. In the case of the phosphorylation of p38 during infection of the HIBCPP cells, infection with the *NmB* strains displayed stronger activation of the MAPK than infection with the *NmC* strains, with the capsule-deficient mutant MC58siaD^−^ causing the strongest activation. As expected, treatment with PMA resulted in activation of Erk1/2 and equally treatment with Anisomycin lead to activation of p38.

### Infection of HIBCPP cells with *N. meningitidis* wild type strains of serogroup B and C is reduced by the inhibition of Erk1/2 and p38 signalling pathways

Next, we were interested in the role of MAPK pathways during infection of HIBCPP cells by *Nm*. Inhibition of the activation of Erk1/2 and p38 signalling was achieved with the help of the specific chemical inhibitors. This was accomplished by incubating HIBCPP cells in the inverted cell culture filter insert system for 1 h with the MAPK/Erk kinase (MEK) 1 and 2 inhibitor U0126, and the inhibitor of p38 activity SB203580, respectively, at a concentration of 25 µM. Phosphorylation of Erk1/2 was strongly decreased by the application of U0126 in the presence of either the *Nm*B strain MC58siaD^−^ or PMA. The phosphorylation of a p38 target, the mitogen-activated protein kinase-activated protein kinase (MAPKAPK) 2, was similarly reduced when cells were stimulated with Anisomycin or infected with the *Nm*B strain MC58siaD^−^ (Additional file 1).

To investigate the influence of these MAPK pathways, infection experiments were conducted with the wild type strains of *Nm*B and *Nm*C. To this end, the HIBCPP cells were pre-incubated with the inhibitors (25 µM each) for 1 h before infection with the *Nm* strains for 4 h (MOI of 10) from the basolateral side. To determine the number of invaded bacteria, double immunofluorescence analysis was used as described in the ﻿“Materials and methods” section.

**Fig. 2:**
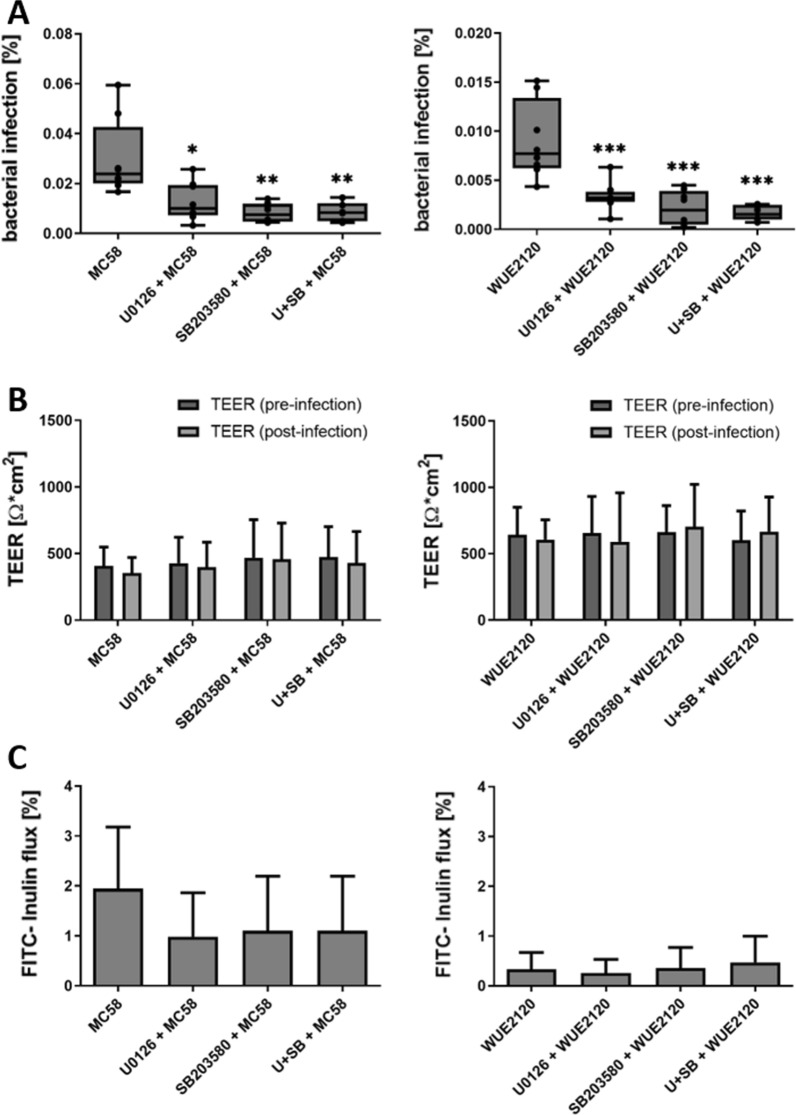
MAPK are required for the infection of the HIBCPP cells by *Nm* wild type strains. HIBCPP cells were incubated for 1 h with the Erk1/2 and p38 inhibitors U0126, SB203580 or both inhibitors (U + SB) at a concentration of 25 μM each. Untreated cells acted as controls. The HIBCPP cells were infected for 4 h at a MOI 10 in the inverted culture. The intracellular bacteria could be quantified by double immunofluorescence staining (**A**). The integrity of the barrier function was determined over the course of the experiment by measuring the TEER values (**B**) and permeability for FITC inulin (**C**). *** extremely significant, p < 0.001; ** highly significant, p < 0.01; * significant, p < 0.05; when comparing the infection rate of treated cells with the infection rate in the control cells

Figure 2A shows that for both strains the inhibition of either Erk1/2 or p38 signalling resulted in a significant reduction of intracellular bacteria in the HIBCPP cells. For both strains, the inhibition of p38 resulted in a stronger reduction of intracellular bacteria than the inhibition of Erk1/2 signalling, whereas the simultaneous inhibition of both pathways (both inhibitors at 25 µM) did not further reduce the number of intracellular bacteria. The TEER values remained stable over the course of the infection experiments in the presence of one or both inhibitors (shown in Fig. 2B). Furthermore, the permeability for the FITC-labelled inulin remained unchanged by the treatment with the inhibitors (shown in Fig. 2C).

### Inhibition of the Erk1/2 signalling pathways displays no inhibitory effect on the invasion of capsule-deficient *Nm*B and *Nm*C in HIBCPP cells during infection, whereas infection of the *Nm*C capsule-deficient mutant is dependent on the MAPK p38

**Fig. 3:**
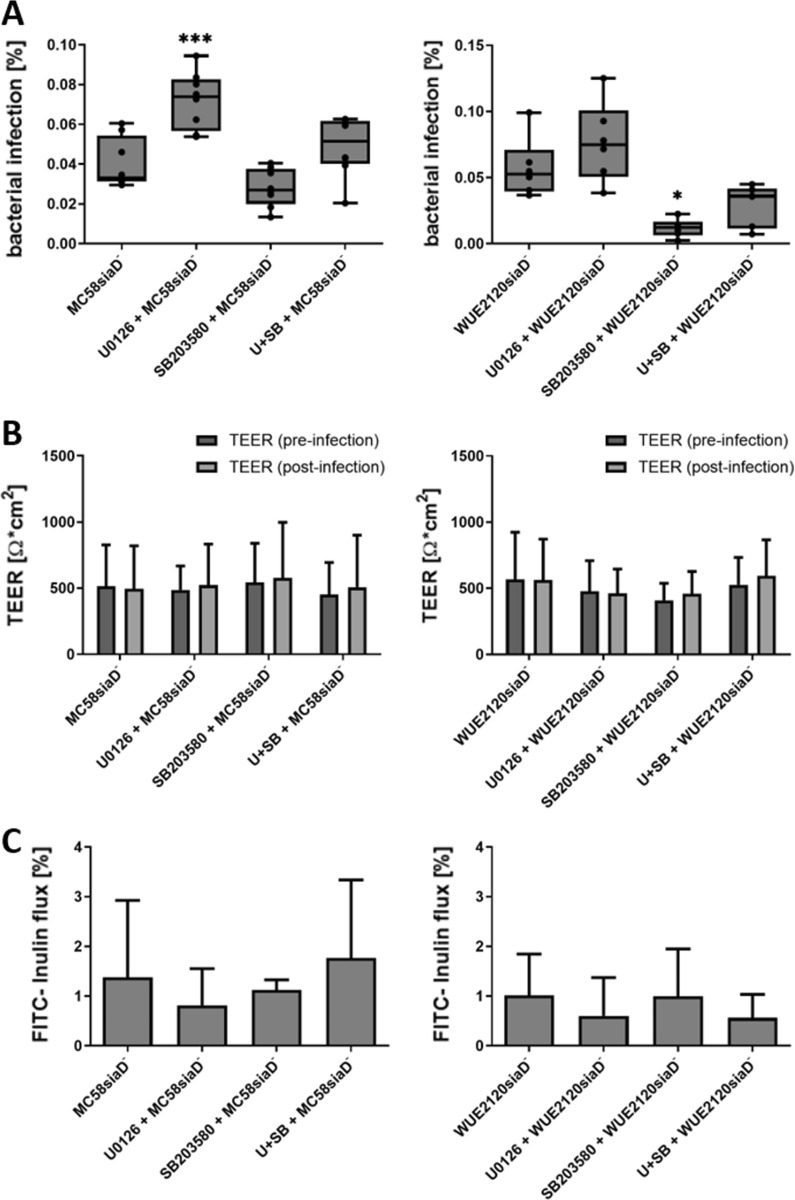
Inhibition of Erk1/2 and p38 signalling impacts infection rates differently in absence of the capsule. The inhibition of the Erk1/2 signalling pathway leads to an increased infection by the capsule-deficient mutants of *Nm*B and *Nm*C, while inhibition of p38 signalling decreases the rate of infection of the *Nm*C capsule-deficient mutant, but has no significant effect on the infection rates of the *Nm*B capsule-deficient mutant. HIBCPP cells were incubated for 1 h with the inhibitors U0126, SB203580 or both inhibitors (U + SB) at a concentration of 25 μM each. Untreated cells acted as controls. The HIBCPP cells were infected for 4 h at a MOI 10 in the inverted culture. The intracellular bacteria could be quantified by double immunofluorescence staining (**A**). The integrity of the barrier function was determined over the course of the experiment by measuring the TEER values (**B**) and permeability for FITC inulin (**C**). *** extremely significant, p < 0.001; ** highly significant, p < 0.01; * significant, p < 0.05; when comparing the infection rate of treated cells with the infection rate in the control cells

To examine if the presence of the capsule, as one of the major virulence factors of *Nm*, has an effect on the infection rates upon inhibition of the Erk1/2 and p38 MAPK pathways, infection experiments were conducted with the capsule-deficient mutant strains of serogroups B and C (MC58siaD^−^ and WUE2120siaD^−^, respectively). The experimental setup remained the same as described for the wild type strains in the previous section. Figure 3A shows that the capsule-deficient mutants did not display reduced amounts of intracellular bacteria upon inhibition of the Erk1/2 pathway, but an increase of intracellular bacteria could be detected for the *NmB* capsule-deficient mutant. The inhibition of the p38 signalling pathway significantly reduced infection rates only for the *NmC* capsule-deficient mutant, while the number of intracellular bacteria remained unchanged for the *NmB* capsule-deficient strain. Simultaneous addition of both inhibitors did not result in significant changes of infection rates for both bacterial strains compared to the untreated controls. The barrier function remained stable over the course of these experiments (shown in Fig. 3B, C).

### Expression profile of HIBCPP cells induced by infection with *Nm* serogroup B

As shown in Figs. 2 and 3, the *Nm*B capsule-deficient mutant displayed unexpected changes in invasion rates after inhibition of the Erk1/2 pathway in comparison to the wild type strain. To determine the impact of Erk1/2 signalling on gene expression during infection of the CP epithelium with *Nm*, the transcriptome of HIBCPP cells after infection with *Nm*B was characterized using a MACE approach. Special emphasis was put on the difference between the wild type strain MC58 and the capsule-deficient mutant MC58siaD^−^. The HIBCPP cells were infected with the two *Nm* strains (MOI 10) for 6 h in the inverted filter culture system in presence and absence of the Erk1/2 inhibitor U0126. RNA was isolated as described and the RIN was determined before the preparation of the libraries, followed by sequencing and bioinformatic processing.

The data of the transcriptome analysis was used to determine differential expression of genes among the different experimental groups. Lists of differentially expressed genes (DEG) were obtained by comparing the gene expression of infected cells with that of the uninfected controls (Additional file 2). In the case of additional treatment with the Erk1/2 signalling inhibitor U0126, the comparisons were drawn between treated controls and treated, infected cells. To assess the differential regulation after treatment and statistical significance of these genes, those lists were filtered using the FDR p-value (p ≤ 0.05) and a minimum log_2_ twofold up- or downregulation.

**Fig. 4:**
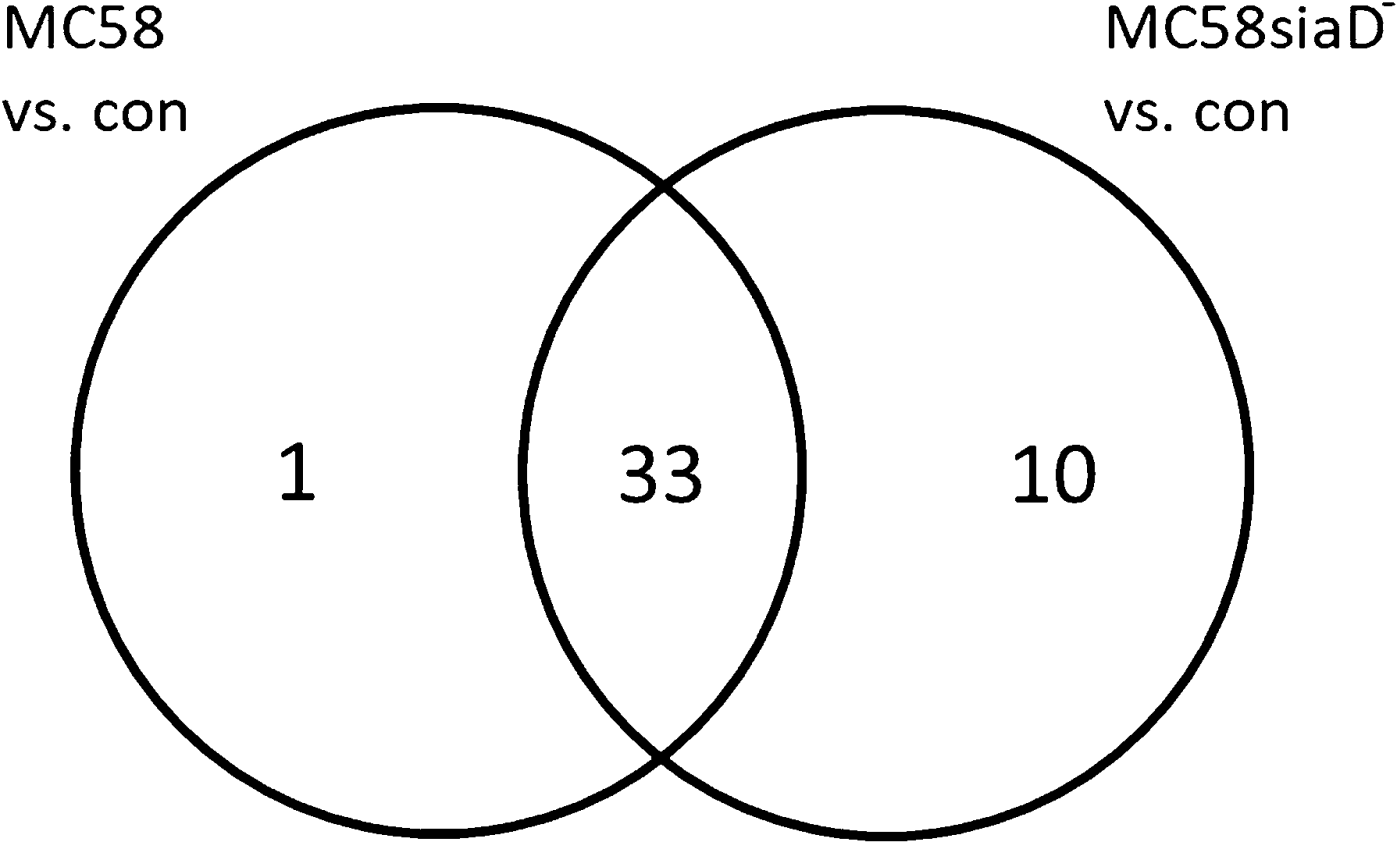
The number of genes, which were regulated in HIBCPP cells after infection with *Nm*B strains. The DEG were determined by comparing the HIBCPP cells infected with the *Nm*B strains with the untreated controls. The DEG, which are regulated in both conditions as well as the DEG, which are only regulated in one of the conditions, are presented in the diagram

After infection of the HIBCPP cells with the *Nm* wild type strain MC58, 34 genes were significantly regulated, 33 of which were also regulated by the capsule-deficient mutant MC58siaD^−^. In addition, infection of the cells with MC58siaD- significantly changed the expression of 10 further genes (shown in Fig. 4).

The strongest upregulation after infection with MC58 was a 64-fold induction of the gene *il1b*, while infection of the HIBCPP cells with MC58siaD^−^ resulted in a 77-fold upregulation of the same gene (Table 2).

**Table 2:** Summary of the DEG regulated by the *Nm*B wild type strain and capsule deficient mutant

Genesymbol	Genname	Fold change	
		con vs. MC58	con vs. MC58siaD^−^
IL1B	Interleukin 1 beta	64.65	77.58
CCL20	C–C motif chemokine ligand 20	23.85	28.72
IL17C	Interleukin 17C	22.48	20.25
CXCL2	C-X-C motif chemokine ligand 2	20.76	19.03
TNF	Tumor necrosis factor	18.47	17.79
IL6	Interleukin 6	0.00	16.49
CXCL1	C-X-C motif chemokine ligand 1	15.80	16.35
CXCL3	C-X-C motif chemokine ligand 3	14.48	14.37
CXCL8	C-X-C motif chemokine ligand 8	14.03	14.45
NFKBIZ	NFKB inhibitor zeta	13.60	11.15
CSF2	Colony stimulating factor 2	13.01	15.08
CCL2	C–C motif chemokine ligand 2	12.47	13.89
IL1A	Interleukin 1 alpha	11.74	11.61
ZC3H12A	Zinc finger CCCH-type containing 12A	11.63	10.14
TCIM	Transcriptional and immune response regulator	9.73	10.53
ICAM1	Intercellular adhesion molecule 1	9.68	9.46
RND1	Rho family GTPase 1	9.04	9.08
CXCL6	C-X-C motif chemokine ligand 6	7.92	9.75
CYP24A1	Cytochrome P450 family 24 subfamily A member 1	7.64	8.70
NFKBIA	NFKB inhibitor alpha	7.46	7.34
TNFAIP2	TNF alpha induced protein 2	7.24	7.81
SPIB	Spi-B transcription factor		6.49
RASD1	ras related dexamethasone induced 1		6.34
IGFBP1	Insulin like growth factor binding protein 1		6.27
IL23A	Interleukin 23 subunit alpha	6.10	8.24
LIF	LIF interleukin 6 family cytokine	5.97	5.01
PTGS2	Prostaglandin-endoperoxide synthase 2	5.89	5.68
ANGPTL4	Angiopoietin like 4	5.69	5.34
ADORA2A	Adenosine A2a receptor		5.44
ZFP36	ZFP36 ring finger protein	5.19	4.48
C6orf222		4.98	
CX3CL1	C-X3-C motif chemokine ligand 1	4.96	4.59
TNFAIP3	TNF alpha induced protein 3	4.94	4.45
CSF1	Colony stimulating factor 1		4.77
PI3	Peptidase inhibitor 3		4.73
SERPINA3	Serpin family A member 3		4.71
IER3	Immediate early response 3	4.60	4.60
SLC6A14	Solute carrier family 6 member 14	4.59	4.97
LTB	Lymphotoxin beta	4.50	5.92
TNFRSF6B	TNF receptor superfamily member 6b		4.44
CEBPD	CCAAT enhancer binding protein delta	4.40	4.47
SOD2	superoxide dismutase 2	4.30	5.21
HILPDA	Hypoxia inducible lipid droplet associated		4.27
MAFF	MAF bZIP transcription factor F	4.21	4.13

Infection with both *NmB* strains displayed very similar expression levels after bacterial challenge for the cytokine genes *cxcl1*, *cxcl2*, *cxcl3, cxcl6* and *ccl2*, independent of the presence or absence of the bacterial capsule. Both *Nm*B strains also induced the expression of *ccl20*. However, infection of HIBCPP cells with the capsule-deficient mutant resulted in a higher fold change compared to infection of the cells with the wild type strain. The interleukin *il17c* and *il1b* genes both displayed a high upregulation after bacterial challenge of the cells. However, infection of HIBCPP cells with the *Nm* wild type strain resulted in higher expression levels of the *il17c* than infection with the capsule-deficient mutant, while, for *il1b*, infection of the HIBCPP cells with the capsule-deficient mutant caused a higher upregulation of the gene than the infection with the wild type strain.

Expression of *nfκbia* and *nfκbiz* were also both significantly upregulated after bacterial challenge. After infection of HIBCPP cells with the *NmB* strains, expression of *nfκbia* displayed similar fold-changes for both wild type strain and capsule-deficient mutant, while *nfκbiz* displayed a stronger upregulation after infection with the wild type strain. Further examples of upregulated genes after challenge with *NmB* are *rnd1*, *tcim*, *tnf* and *zc3h12a* as well as the intercellular adhesion molecule *icam1*.

Overall, the analysis of the expression profile of the HIBCPP cells after challenge with the *NmB* strains showed that the capsule-deficient mutant displayed a higher number of DEG under the parameters of the statistical analysis than infection of HIBCPP cells with the wild type strain.

### Influence of the inhibition of the Erk1/2 pathway on the gene expression after bacterial challenge of HIBCPP cells with *NmB* wild type and capsule-deficient mutant

**Fig. 5:**
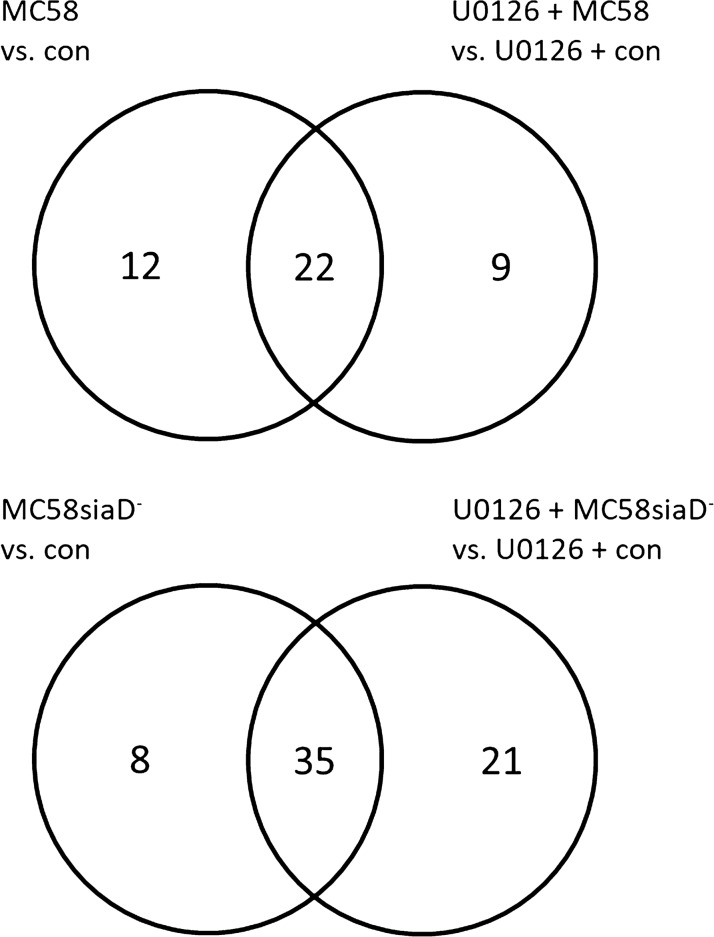
Number of genes regulated in HIBCPP cells after Erk1/2 treatment and infection with *Nm*B strains. The DEG were determined by comparing the HIBCPP cells infected with the *Nm*B strains with the untreated controls as well as the treated control cells with the treated, infected cells. Shown are the DEG, which are regulated in both conditions, and the DEG, which are only regulated in one of the conditions

Furthermore, genes were identified, which were significantly regulated by infection with the *Nm*B strains after pretreatment of the HIBCPP cells with the inhibitor U0126 (shown in Fig. 5). A total of 31 DEG could be determined after infection with the wild type strain in presence of the inhibitor, when compared to the uninfected control. 22 of these genes were also regulated following infection of HIBCPP cells in absence of U0126. Interestingly, 9 genes could be identified, which were significantly regulated after infection with MC58 only if the HIBCPP cells had previously been treated with U0126. 12 genes are only regulated by *Nm*B in absence of Erk1/2 inhibition.

During infection of HIBCPP cells with the capsule-deficient mutant MC58siaD^−^, a total of 35 DEG were determined, which were regulated in the presence and absence of the inhibitor U0126 (shown in Fig. 5). An additional 21 DEG could be determined, which were only significantly induced when the Erk 1/2 signalling pathway was inhibited during the infection of the HIBCPP cells, while 8 DEG were only upregulated in the absence of the inhibitor.

When comparing the DEG of HIBCPP cells after infection with the *Nm*B wild-type strain in presence and absence of the Erk1/2 inhibitor U0126 (Table 3), the determined fold changes are almost identical. An exception is *il17c*, which is upregulated 22-fold after infection with the *Nm*B wild type strain in absence of U0126, but is upregulated 79-fold by the bacteria when the Erk1/2 signalling pathway is inhibited. *il8* also displayed a higher upregulation by *Nm*B after infection of pre-treated cells than after infection of untreated HIBCPP cells. In contrast, the expression levels of the genes *ccl20*, *icam1* and *nfκbiz* displayed higher fold changes induced by *Nm*B when Erk1/2 signalling was not inhibited.

**Table 3:** Summary of the DEG regulated by the *Nm*B wild type strain in presence and absence of the inhibitor U0126

Genesymbol	Genname	Fold change	
		con vs. MC58	con + U0126 vs. MC58 + U0126
IL1B	INTERLEUKIN 1 beta	64.65	
CCL20	C–C motif chemokine ligand 20	23.85	20.68
IL17C	Interleukin 17C	22.48	79.66
CXCL2	C-X-C motif chemokine ligand 2	20.76	20.30
TNF	Tumor necrosis factor	18.47	18.21
CXCL1	C-X-C motif chemokine ligand 1	15.80	16.79
CXCL3	C-X-C motif chemokine ligand 3	14.48	15.66
CXCL8	C-X-C motif chemokine ligand 8	14.03	16.17
NFKBIZ	NFKB inhibitor zeta	13.60	8.18
CSF2	colony stimulating factor 2	13.01	
CCL2	C–C motif chemokine ligand 2	12.47	11.80
IL1A	Interleukin 1 alpha	11.74	
ZC3H12A	Zinc finger CCCH-type containing 12A	11.63	12.04
ANKRD37	Ankyrin repeat domain 37		10.05
TCIM	Transcriptional and immune response regulator	9.73	6.77
ICAM1	Intercellular adhesion molecule 1	9.68	7.98
RND1	Rho family GTPase 1	9.04	7.02
CXCL6	C-X-C motif chemokine ligand 6	7.92	7.67
CYP24A1	Cytochrome P450 family 24 subfamily A member 1	7,64	
NFKBIA	NFKB inhibitor alpha	7.46	7.33
TNFAIP2	TNF alpha induced protein 2	7.24	5.59
IL23A	Interleukin 23 subunit alpha	6.10	
LIF	LIF interleukin 6 family cytokine	5.97	
PTGS2	Prostaglandin-endoperoxide synthase 2	5.89	
ADORA2A	Adenosine A2a receptor		6.91
ADM	Adrenomedullin		6.36
PFKFB4	6-phosphofructo-2-kinase/fructose-2,6-biphosphatase 4		6.34
ANGPTL4	Angiopoietin like 4	5.69	7.59
MIR210HG	MIR210 host gene		5.63
IL4I1	Interleukin 4 induced 1		5.48
CXCL10	C-X-C motif chemokine ligand 10		5.40
ZFP36	ZFP36 ring finger protein	5.19	
C6orf222		4.98	5.77
CX3CL1	C-X3-C motif chemokine ligand 1	4.96	6.12
TNFAIP3	TNF alpha induced protein 3	4.94	5.03
FBXL8	F-box and leucine rich repeat protein 8		4.75
BHLHE40	Basic helix-loop-helix family member e40		4.36
IER3	IMMEDIATE early response 3	4.60	
SLC6A14	Solute carrier family 6 member 14	4.59	
LTB	Lymphotoxin beta	4.50	
CEBPD	CCAAT enhancer binding protein delta	4.40	4.01
SOD2	Superoxide dismutase 2	4.30	4.09
MAFF	MAF bZIP transcription factor F	4.21	

The DEG summarized in Table 4 demonstrate that the inhibition of the Erk1/2 signalling pathway during infection of the HIBCPP cells with the *Nm*B capsule-deficient mutant resulted in similar or, in most cases, a stronger upregulation of gene expression by the bacteria than in infected, untreated cells. *il17c*, which displayed a 20-fold upregulation during infection of untreated HIBCPP cells with the *Nm* capsule-deficient mutant, was upregulated 191-fold by the bacteria when the Erk1/2 signalling pathway was inhibited during infection. Exceptions are the genes *cyp24a1*, *il1b*, *il23a*, *il6*, *nfκbiz* and *ptgs2*, which display a lower fold-change induced by MC58siaD^−^ after pre-treatment with the inhibitor. The expression of *il1b*, which had the strongest upregulation in untreated cells after infection with both *Nm*B strains (65-fold by the wild type, 78-fold by MC58saiD^−^), is upregulated only 57-fold by MC58saiD^−^ when Erk1/2 signalling was inhibited during infection. 7 genes could be identified that were specifically induced by both the *Nm*B wild type strain and the capsule-deficient mutant only after pre-treatment with the inhibitor U0126. These genes are *adm*, *ankrd37*, *bhlhe40*, *cxcl10*, *il4i1*, *mir210hg*, and *pfkfb4*. Infection of HIBCPP cells with the capsule-deficient mutant resulted in upregulation of additional 14 genes specifically during treatment with U0126, which were not regulated by the wild type strain under the same conditions, including *plau*, *nos2* and *tnfrsf11b*.

**Table 4:** Summary of the DEG regulated by the *Nm*B capsule-deficient mutant in presence and absence of the inhibitor U0126

Genesymbol	Genname	Fold change	
		con vs. MC58siaD^−^	con + U0126 vs. MC58siaD^−^ + U0126
IL1B	Interleukin 1 beta	77.58	56.80
CCL20	C–C motif chemokine ligand 20	28.72	37.32
IL17C	INTERLEUKIN 17C	20.25	190.68
CXCL2	C-X-C motif chemokine ligand 2	19.03	26.60
TNF	Tumor necrosis factor	17.79	26.58
IL6	interleukin 6	16.49	8.56
CXCL1	C-X-C motif chemokine ligand 1	16.35	25.62
CSF2	colony stimulating factor 2	15.08	15.09
CXCL8	C-X-C motif chemokine ligand 8	14.45	26.67
CXCL3	C-X-C motif chemokine ligand 3	14.37	21.85
CCL2	C–C motif chemokine ligand 2	13.89	22.91
ANKRD37	Ankyrin repeat domain 37		12.78
IL1A	Interleukin 1 alpha	11.61	28.19
NFKBIZ	NFKB inhibitor zeta	11.15	8.80
TCIM	Transcriptional and immune response regulator	10.53	10.40
ZC3H12A	Zinc finger CCCH-type containing 12A	10.14	14.27
CXCL6	C-X-C motif chemokine ligand 6	9.75	13.31
IL4I1	Interleukin 4 induced 1		9.49
ICAM1	Intercellular adhesion molecule 1	9.46	11.60
RND1	Rho family GTPase 1	9.08	11.45
CXCL10	C-X-C motif chemokine ligand 10		8.81
CYP24A1	Cytochrome P450 family 24 subfamily A member 1	8.70	4.63
NOS2	Nitric oxide synthase 2		8.64
IL23A	Interleukin 23 subunit alpha	8.24	7.28
TNFAIP2	TNF alpha induced protein 2	7.81	8.00
SAA2	Serum amyloid A2		7.67
C6orf222			7.67
GFPT2	Glutamine-fructose-6-phosphate transaminase 2		7.60
PFKFB4	6-phosphofructo-2-kinase/fructose-2,6-biphosphatase 4		7.43
NFKBIA	NFKB inhibitor alpha	7.34	9.29
ADM	Adrenomedullin		7.28
MIR210HG	MIR210 host gene		6.90
SPIB	Spi-B transcription factor	6.49	
CHAC1	ChaC glutathione specific gamma-glutamylcyclotransferase 1		6.48
RASD1	Ras related dexamethasone induced 1	6.34	
IGFBP1	Insulin like growth factor binding protein 1	6.27	
ANKK1	Ankyrin repeat and kinase domain containing 1		5.99
LTB	Lymphotoxin beta	5.92	5.95
PTGS2	Lymphotoxin beta	5.68	4.50
G0S2	G0/G1 switch 2		5.46
ADORA2A	Adenosine A2a receptor	5.44	11.67
ZC3H12C	Zinc finger CCCH-type containing 12C		5.44
ANGPTL4	Angiopoietin like 4	5.34	10.50
LOC101929444			5.29
SOD2	Superoxide dismutase 2	5.21	7.17
LIF	LIF interleukin 6 family cytokine	5.01	
SLC6A14	SOLUTE carrier family 6 member 14	4.97	6.04
TNFRSF11B	TNF receptor superfamily member 11b		4.90
CSF1	Colony stimulating factor 1	4.77	
PI3	Peptidase inhibitor 3	4.73	4.62
SERPINA3	Serpin family A member 3	4.71	6.36
EGLN3	egl-9 family hypoxia inducible factor 3		4.70
IER3	Immediate early response 3	4.60	
CX3CL1	C-X3-C motif chemokine ligand 1	4.59	8.78
PFKFB3	6-phosphofructo-2-kinase/fructose-2,6-biphosphatase 3		4.59
RCAN1	Regulator of calcineurin 1		4.54
ZFP36	ZFP36 ring finger protein	4.48	
CEBPD	CCAAT enhancer binding protein delta	4.47	4.97
TNFAIP3	TNF alpha induced protein 3	4.45	5.48
TNFRSF6B	TNF receptor superfamily member 6b	4.44	7.26
BHLHE40	Basic helix-loop-helix family member e40		4.38
HILPDA	Hypoxia inducible lipid droplet associated	4.27	4.99
PLAU	Plasminogen activator, urokinase		4.02
MAFF	MAF bZIP transcription factor F	4.13	

### Confirmation of the MACE data by QPCR analysis

The obtained data was validated by verifying the expression levels of selected genes using qPCR. Basis for the selection of these genes were the filtered lists of DEGs presented in Tables 2, 3, 4. For the validation of the data obtained for gene expression after infection with the *NmB* and *NmC* strains in presence and absence of the capsule, *il6*, *il8*, *nfkbiz*, *zc3h12a* and *tnf* were chosen. These genes were chosen as they were previously shown to be regulated in HIBCPP cells after infection with *Nm* by Borkowski et al. [4].

Table 5 summarizes the fold changes and the corresponding FDR p-values of the selected genes during the MACE analysis compared to the relative fold changes (2^−ΔΔCT^) obtained by the qPCR analysis of uninfected versus infected cells along with the standard deviations. Overall, the results obtained by the MACE analysis could be verified via qPCR for the chosen genes.

**Table 5:** Validation of the MACE data by qPCR

	qPCR	Stdev	MACE	FDR p-value
NFκβIZ				
Con vs. MC58	13.43	0.98	13.60	0.00
Con + U0126 vs. MC58 + U0126	13.17	2.87	8.18	0.00
Con vs. MC58siaD^−^	11.16	3.79	11.15	0.00
Con + U0126 vs MC58siaD^−^ + U	11.99	4.21	8.80	0.00
Zc3H12A				
Con vs. MC58	11.98	2.54	11.63	0.00
Con + U0126 vs. MC58 + U0126	11.28	2.91	12.04	0.00
Con vs. MC58siaD^−^	11.81	2.83	10.14	0.00
Con + U0126 vs MC58siaD^−^ + U	10.40	2.35	14.27	0.00
TNFα				
Con vs. MC58	14.35	2.89	18.47	0.00
Con + U0126 vs. MC58 + U0126	17.08	1.72	18.21	0.00
Con vs. MC58siaD^−^	15.36	2.55	17.79	0.00
Con + U0126 vs MC58siaD^−^ + U	22.21	3.53	26.58	0.00
IL6				
Con vs. MC58	11.52	3.58	-	-
Con + U0126 vs. MC58 + U0126	4.28	0.95	-	-
Con vs. MC58siaD^−^	16.16	5.62	16.49	0.02
Con + U0126 vs MC58siaD^−^ + U	7.73	3.32	8.56	0.02
IL8				
Con vs. MC58	18.21	8.86	14.03	0.00
Con + U0126 vs. MC58 + U0126	30.36	8.88	16.17	0.00
Con vs. MC58siaD^−^	19.51	6.04	14.45	0.00
Con + U0126 vs MC58siaD^−^ + U	61.58	33.68	26.67	0.00

The relative fold changes were determined using the 2^−ΔΔCT^ method. The mean values and standard deviations (Stdev) of the qPCR data as well as the fold change of the DEG and the FDR p-values are shown

### Gene ontology (GO) term analysis of HIBCPP cells infected with *Nm*B strains in presence and absence of the Erk1/2 inhibitor U0126

To gain more insight on the molecular function of the genes regulated during infection, we performed a GO analysis. To this end, the GO software LAGO was used for identification of statistically overrepresented GO terms. The lists of DEG used for the GO analysis can be found as supplemental data (Additional file 3). The analysis was performed using a p-value cut-off of 0.01. A selection of the most overrepresented GO Terms is given in Table 6. During infection of HIBCPP cells with the *NmB* strains, the most significantly overrepresented GO terms are pointing towards a role of the CP epithelial cells in the inflammatory response. These include the terms “cellular response to lipopolysaccharide”, “cellular response to molecule of bacterial origin” and “cytokine-mediated signalling pathway”. The regulation of cellular signalling pathways during infection was described by terms such as " NIK/NF-kappaB signalling” during infection with both *Nm*B strains, as well as a regulation of the MAPK signalling pathways, represented by the terms “positive regulation of MAPK cascade” and the “regulation of ERK1 and ERK2 cascade”. The GO terminus, describing the negative regulation of IL6 production ("negative regulation of interleukin-6 production"), was overrepresented for both *Nm*B strains. Another GO term, which was overrepresented during the infection with both *Nm*B strains, describes the “negative regulation of cell death”.

**Table 6:** Statistically overrepresented GO terms

GO Term	Con vs MC58		Con vs MC58siaD		Con + U0126 vs MC58 + U0126		Con + U 0126 vs MC58siaD + U0126	
	p-value	Number annotated	p-value	Number Aannotated	p-value	Number annotated	p-value	Number annotated
Cellular response to lipopolysaccharide	1.64 × 10^–20^	16	3.03 × 10^–20^	17	1.49 × 10^–15^	13	1.35 × 10^–19^	18
Cellular response to molecule of bacterial origin	3.39 × 10^–20^	16	6.55 × 10^–20^	17	2.68 × 10^–15^	13	3.06 × 10^–19^	18
Cytokine-mediated signalling pathway	4.08 × 10^–20^	22	2.22 × 10^–21^	25	5.17 × 10^–12^	16	6.30 × 10^–19^	26
Cellular response to interleukin-1	1.97 × 10^–08^	9	6.48 × 10^–09^	10	1.08 × 10^–05^	7	1.16 × 10^–07^	10
Regulation of NIK/NF-kappaB signalling	4.63 × 10^–05^	6	2.26 × 10^–04^	6			1.21 × 10^–03^	6
NIK/NF-kappaB signalling	5.84 × 10^–04^	6	2.78 × 10^–03^	6				
Interleukin-6 production	1.93 × 10^–04^	6	3.37 × 10^–05^	7			1.00 × 10^–05^	8
Negative regulation of interleukin-6 production	9.11 × 10^–04^	4	2.68 × 10^–03^	4	4.99 × 10^–04^	4	8.35 × 10^–03^	4
Cell surface receptor signalling pathway	1.82 × 10^–10^	25	7.24 × 10^–12^	30	1.73 × 10^–06^	20	3.69 × 10^–09^	32
Signal transduction by protein phosphorylation	3.11 × 10^–03^	10	4.96 × 10^–03^	11				
MAPK cascade	2.85 × 10^–03^	10	4.51 × 10^–03^	11				
Positive regulation of MAPK cascade	3.93 × 10^–03^	8	2.99 × 10^–03^	9				
Regulation of ERK1 and ERK2 cascade	7.13 × 10^–04^	7	4.27 × 10^–03^	7				
JAK-STAT cascade	8.91 × 10^–03^	5	1.56 × 10^–03^	6				
Negative regulation of cell death	2.99 × 10^–08^	15	6.62 × 10^–11^	19	1.79 × 10^–04^	11	1.76 × 10^–06^	17
Response to tumour necrosis factor	9.23 × 10^–11^	12	9.21 × 10^–11^	13	8.05 × 10^–07^	9	4.24 × 10^–09^	13
Regulation of tumour necrosis factor production	3.14 × 10^–04^	6	5.93 × 10^–05^	7			7.86 × 10^–03^	6
Regulation of tumour necrosis factor superfamily cytokine production	3.52 × 10^–04^	6	6.78 × 10^–05^	7			8.79 × 10^–03^	6

The GO terms were determined on the basis of the different lists of DEG. The analysis was carried out with the LAGO software and an upper limit of 0.01 was applied as p-value with a Bonferroni correction. The GO terms, the corrected p-value and the number of analysed genes in the respective GO terms (number of genes) are shown

The GO termini identified during *Nm* infection of HIBCPP cells pre-treated with the Erk1/2 inhibitor U0126 revealed that, similar to untreated cells, GO terms indicating a role of the CP epithelial cells in the inflammatory response were overrepresented. Some of the GO terms that had been identified after infection of the nontreated cells were no longer found after inhibition of Erk1/2. These included GO termini indicative of manipulation of MAPK signalling pathways (“MAPK cascade”, “positive regulation of MAPK cascade”, “regulation of ERK1 and ERK2 cascade”), underscoring the function of the inhibitor during the experiments. Furthermore, the GO terms "signal transduction by protein phosphorylation" and "JAK-STAT cascade" were no longer identified.

Differences from the non-treated cells were also identified in terms of under-representation of GO termini suggestive of involvement of the NF-κB signalling pathway. Whereas the GO term “NIK/NF-κB signalling” was not identified following infection with both strains, the related term “regulation of NIK/NF-κB signalling” was still overrepresented following infection with MC58siaD^−^, but not with the wild type. Further GO terms, which in U0126 treated cells were only identified following infection with the mutant are “regulation of interleukin-6” production and GO terms indicative of a role of tumour necrosis factor (“regulation of tumour necrosis factor production”, “regulation of tumour necrosis factor superfamily cytokine production”).

## Discussion

To cause diseases, such as meningitis, *Nm* needs to cross host cell barriers, among which are the BBB and the BCSFB. In order to enter the CNS via the BCSFB, the pathogens have to overcome the epithelial cells of the CP [21, 22]. *Nm* have been demonstrated to interact with the CP epithelium, which constitutes the morphological correlate of the BCSFB, during infection [3, 4, 8, 32, 33]. The CP epithelial cells not only present a barrier function against invading pathogens, they also detect bacterial pathogens and, in response, generate a variety of mediators that function in the activation of innate and adaptive immunity [34].

Many pathogens have been demonstrated to target cell signalling pathways in order to inhibit or modulate the immune response of the host [35]. The MAPK signalling pathways convert extracellular signals into cellular responses, which, upon challenge with a pathogen, are activated and lead to a subsequent expression of inflammatory cytokines and chemokines [36, 37]. Furthermore, MAPK function can be exploited by pathogens to their benefit [37]. However, the role of MAPK during infection with *Nm* is still largely unknown, especially with regards to the BCSFB*.*

We determined a moderate activation of Erk1/2 in response to the infection with *Nm*. This stands in contrast to the infection of the HIBCPP cells with *Lm*, which induced a stronger activation of Erk1/2 [14]. The MAPK p38 on the other hand, displayed a pronounced activation by all *Nm* strains of serogroups B and C, with a stronger phosphorylation being observed after infection with the *Nm*B strains. In HBMEC, an in vitro cell culture system of the BBB, activation of MAPK p38 and JNK after infection with the *Nm*B wild type strain MC58 and its capsule-deficient mutant has already been described [15]. However, we did not observe an activation of JNK after infection with *Nm* (data not shown), indicating differential involvement of MAPK pathways at the BBB and BCSFB.

To analyse the role of the two MAPK signalling pathways during the infection of HIBCPP cells with *Nm*, the cells were pre-treated with specific inhibitors that prevent the activation of Erk1/2 (U0126) and p38 (SB203580). Both inhibitors have a high selectivity, which has been proven in several studies [38, 39]. The inhibition of the Erk1/2 signalling during infection of HIBCPP cells resulted in a significant reduction of the intracellular bacteria of the *Nm*B and *Nm*C wild type strains. This correlates with the results of Dinner et al., which describe a dependence of the infection of *Lm* in HIBCPP cells on Erk1/2 signalling [14]. In HBMEC a role of the p38 and JNK signalling pathways during infection with *Nm* could be observed, however not the activation of Erk1/2 [15], again pointing to specific activation of MAPK pathways at the BCSFB and BBB, respectively.

In order to investigate the influence of the polysaccharide capsule under the same experimental conditions, infections of HIBCPP cells were also carried out with the capsule-deficient mutants of the *Nm* strains. Both capsule-deficient mutants of the *Nm*B and NmC strains displayed an increased number of intracellular bacteria in HIBCPP cells, with only MC58siaD^−^ displaying a significant increase of infection rates, after inhibition of the Erk1/2 signalling pathway. This is in stark contrast to the wild-type strains and suggests that the capsule of the pathogen may cover important virulence factors that can influence the infection mechanism of the pathogen. We previously detected capsule-dependent endocytosis mechanisms of *Nm* in HIBCPP cells involving actin related protein 2/3 (Arp2/3) and possibly dynamin, which could further impact downstream signalling [40]. It requires further investigation to determine to which extend MAPKs influence these mechanisms. Lower infection rates of the *Nm*B and *Nm*C wild type strains compared to the capsule-deficient mutants have already been described [3, 4] and could also be confirmed in this work [41, 42]. In addition, in a study using macrophages infected with the Gram-negative bacterium *Burkholderia pseudomallei*, no effect on the number of intracellular bacteria was found after inhibition of the Erk1/2 signalling pathway compared to untreated, infected cells [43]. Interestingly, clinical application of Erk inhibitors has been suggested in the context of cancer therapy and treatment of neurological disorders as well as being discussed as a target during viral infections [44–47]. Furthermore, inhibition of Erk signalling has resulted in exacerbated SARS-CoV-2 infection in vitro [48], underscoring the importance of understanding the differential regulation of this signalling pathway during infection.

Both wild type strains of the *Nm* serogroups B and C displayed significantly reduced infection rates in HIBCPP cells after p38 inhibition. However, a significant reduction in the number of intracellular bacteria of the *Nm*C capsule-deficient mutant in HIBCPP cells could be observed, but no significant change in the infection rates for the *Nm*B capsule-deficient mutant could be determined after inhibition of p38 signalling. The combination of both inhibitors resulted in a significant decrease of infection rates for the *Nm* wild type strains, but no significant change in infection rates could be observed for the capsule-deficient mutants. Taken together, the role of Erk1/2 and p38 signalling differs during infection of HIBCPP cells by wild type and capsule-depleted *Nm*, with inhibition of Erk1/2 leading to rather opposite effects on wild type and mutant bacteria.

The release of cytokines and chemokines from structures protecting the CSF in response to infection is of special importance as the CSF presents an immunologically privileged site. The recognition and elimination of pathogens is another crucial step during host defence and can result in further release of signalling molecules [32, 49]. In this context, the onset of inflammation acts as an unspecific mechanism early on in response to an infection to limit the spread of the pathogen as well as activating and attracting immune cells [37]. It is known that activation of signalling events induced by bacteria is linked to the modulation of the cytokine response of infected macrophages and epithelial cell lines [14, 15, 50–53]. Among other mechanisms, the MAPK are involved in the induction and regulation of the inflammatory response [37], and pathogens can modulate the inflammatory response in host cells and induce anti-inflammatory cytokines by selective Erk1/2 signalling [54].

The induction of proinflammatory cytokines and chemokines during infection of porcine and human CP cells with bacteria has previously been demonstrated in our laboratory [4, 55, 56]. Here, we applied the MACE RNA-seq technology, which sequences only the 3` end of the transcript and enables sequencing of samples at a deeper level. This is achieved by each read being representative of only one transcript in the sample [57]. The dataset generated from HIBCPP cells infected from the physiologically relevant basolateral side with *NmB* wild type strain and capsule-deficient mutant in presence and absence of Erk1/2 inhibition was evaluated on the differential expression of individual genes as well as biologically interpreted by gene ontology analysis between uninfected and infected cells. We detected a higher number of DEGs following infection with the capsule capsule-deficient mutant compared to infection with the wild type strain. This agrees with previously published data, where a capsule-dependent influence on gene expression after infection with *Nm* has already been shown [41, 42].

During a bacterial meningitis, TNFα and IL1 can be detected in CSF [58]. In addition, IL6 is often found in the CSF after infections with Gram-negative bacteria. The HIBCPP cells infected with *Nm*B displayed a high induction of *il1β* as well as significantly increased expression of *tnf*. The induction of *il6* after bacterial challenge could not be demonstrated in all tested conditions.

Other cytokines known to be upregulated in the CSF during meningitis are CSF2 (GM-CSF), CCL2 (MCP-1), CCL4 (MIP-1β) [58–60]. The gene *csf2* was significantly upregulated after infection with the *Nm*B wild type strain as well as during the infection with the *Nm*B capsule-deficient mutant when Erk1/2 signalling was inhibited. *Ccl2* was found to be significantly upregulated in all examined conditions, but differential expression of *ccl4* could not be determined. Relatively high concentrations of MCP-1 and also of IL6 could be detected in meningioma cells after infection with *Nm*. However, other cytokines and chemokines, such as TNFα, IL1β, CCL3 (MIP-1α), CCL4, CSF2, CSF3 (G-CSF) and CSF1 (M-CSF), were only found in low concentrations in these cells [61]. Interestingly, anti-inflammatory cytokines detected in the CSF of meningitis patients such as IL10, TGFβ or the IL1 receptor antagonists IL1Rs and IL1R2 [58] could not be detected in this study, suggesting that the CP epithelium might not be the source of these factors during meningitis.

The expression of chemokines is regulated by NF-κB signalling, which is induced during infection [62, 63]. A microarray-based transcriptome analysis of HIBCPP cell infection with wild type and capsule-deficient mutant *NmB* hinted towards an NFκB-mediated pro-inflammatory immune response that involved up-regulation of the transcription factor IκBζ [4]. Accordingly, we found upregulation of *nfκbiz*, the gene encoding for IκBζ, following infection. Further genes pointing towards NF-κB signalling during infection, such as *il1b*, *il23a*, *nfκbia*, *tcim* and *zc3h12a*, were also found to be induced by the bacteria in this study in presence or absence of the Erk1/2 inhibitor. The secretion of the CXC chemokines CXCL1-3 was detected in the CP of rats in the blood (apical) and in the CSF (basolateral) [64]. These pro-inflammatory cytokines and chemokines were also found to be upregulated after infection of HIBCPP cells with both *Nm*B strains. Furthermore, increased concentrations of CXCL1-3 and IL8 (CXCL8) were found in meningitis patients [65]. In the MACE analysis, we found also *il8* upregulated in HIBCPP cells after infection with both *NmB* strains. In agreement, while IL8 is hardly detectable in the CSF in healthy individuals, a significant increase in the chemokine has been demonstrated in various studies in the CSF of patients with bacterial meningitis [62].

Furthermore, it has been shown that the chemokine *ccl20* is more strongly expressed in the CP than in other regions of the brain [66]. After infection with the *Nm* strains, *ccl20* was found among the gene products most highly induced by the bacteria, even after inhibition of Erk1/2 signalling. An increased expression of *ccl20* was observed in HBMEC after infection with *Salmonella tymphimurium*. Patients with pneumococcal meningitis also displayed an increased concentration of the chemokine [67, 68].

Another gene upregulated after infection of HIBCPP cells is TCIM which is often associated with a lethal course of cancer [70]. TCIM is also upregulated by the pro-inflammatory cytokines TNFα and IL1β [71] and has been assigned a regulatory role during inflammation in endothelial cells [70]. Furthermore, RND1 is upregulated in infected HIBCPP cells, which plays roles during cell adhesion, modulation of the actin cytoskeleton, and viral infections [72, 73]. The induction of the expression of TCIM and RND1 was also previously determined after infection of HIBCPP cells with *Nm*B [4, 74].

ICAM-1, a cell surface glycoprotein which has been implicated in leukocyte transendothelial migration, is up-regulated in response to inflammatory stimulation. Furthermore, ICAM-1, which was differentially upregulated in HIBCPP cells in all examined conditions after infection, also serves as a signalling receptor transducing outside signalling such as leukocyte adhesive interactions with intracellular epithelial and endothelial function [79]. Signalling cascades downstream of ICAM include MAPK and Src kinase, both of which have been implicated in infection with *Nm* [4, 40, 79] as well as impacting barrier function of epithelial cells. Due to the diverse functions of ICAM, it has been discussed as a promising prognostic biomarker, and a potential target for emerging therapies [79].

The MACE analysis identified several genes that were regulated after infection with *Nm*B specifically in the absence or presence of the inhibitor U0126. In the case of Erk1/2 inhibition after infection with the wild type strain, nine regulated genes were identified, of which seven were also regulated after infection with the capsule-deficient mutant when Erk1/2 was inhibited. Some of the seven genes are involved in the inflammatory response, including CXCL10, IL4I1, and Adrenomedullin (ADM). ADM helps maintain vascular integrity and stabilizes the BBB, possibly through a regulatory effect on the expression of claudin 5 [75]. An increased expression of ADM was found in HBMEC and HIBCPP cells after infection, as well as in plexus carcinoma cells [4, 67, 76]. ADM is of particular interest because it has an anti-microbial effect against Gram-positive and Gram-negative bacteria [77, 78] and its release presumably combats the pathogenic agents. In addition, it was observed in vivo that ADM can bind complement factor H, a negative regulator of the complement cascade. The resulting inhibition of complement activation protects the brain from damage [75]. The release of ADM can also activate G-protein-coupled receptors, which act via cAMP/PKA signalling pathways. This results in a reduction of pro-inflammatory cytokines, as well as an increased expression of anti-inflammatory cytokines and a reduced activation of immune cells [77]. Interestingly, the capsule-deficient mutant specifically regulates 14 further genes in case of Erk1/2 inhibition, including *nos2,* *tnfrsf11b* and the urokinase-type plasminogen activator (PLAU), which belongs to the PLAU receptor (PLAUR) system. PLAU has a pro-inflammatory effect and was detected in the CSF of patients with bacterial meningitis [69]. Overall, our gene expression data largely recapitulated previously detected gene expression following infection with *Nm*, including the described upregulation of *nfκbiz* and *il6* [4]. The specific regulation of genes dependent on active or inactive Erk1/2 signalling points out that MAPKs impact the host cell response at the CP epithelium during infection with *Nm*. Similarly, importance of the p38 MAPK pathway for the control of interleukin-6 (IL-6) and IL-8 has been shown previously following infection of HBMEC with *Nm* [15]. Further studies are needed to analyse to which extend specifically regulated genes play a role during Erk1/2 regulation of HIBCPP cell infection by *Nm*.

The analysis of GO terms pointed towards an inflammatory response of the HIBCPP cells after infection with *Nm*, confirming previous transcriptome data of PC epithelial cells in response to bacterial infection [3, 4, 56]. Furthermore, GO terms indicating a negative regulation of apoptosis as well as a cellular response to TNF were detected in all examined conditions. For invasive ST-11 isolates of *Nm*, a TNFα dependent induction of apoptosis was described in epithelial cells, whereas an apoptosis-reducing effect was found for non-invasive carrier isolates [80].

The GO term analysis confirmed the effect of the Erk1/2 inhibitor U0126, since GO terms describing a regulation of the MAPK signalling cascades were only detected when the DEG of the untreated conditions were analysed. In addition, the terms “signal transduction by protein phosphorylation” and “JAK-STAT cascade” were not detected in untreated conditions.

Interestingly, after Erk1/2 inhibition and infection of HIBCPP cells with the *Nm*B strains, GO termini indicating an involvement of the NF-κB signalling pathway, a function of IL6, and a role of the tumour necrosis factor, were underrepresented compared to the untreated cells. It remains to be determined which role these cellular functions play in regulating the infection of HIBCPP cells by the MAPK signalling pathways. This underrepresentation of GO termini was more pronounced after infection with the wild type strain, which could explain the differential impact of U0126 on infection of HIBCPP cells by the wild type and the capsule-deficient mutant. The GO terms “regulation of NIK / NFκB signalling” and “interleukin-6 production” were determined for Erk1/2 inhibition only after infection with the capsule-deficient mutant, but not the wild-type strain. Activation of NFκB in HIBCPP cells and NFκB-induced expression of inflammatory reaction genes, including *il6*, after infection with *Nm* has already been shown [4]. Furthermore, the GO terms “regulation of tumour necrosis factor production” and “regulation of tumour necrosis factor superfamily cytokine production” were identified in the case of Erk1/2 inhibition only after infection with the capsule-deficient mutant. The role of these processes during infection of HIBCPP cells by *Nm*B requires further investigation.

**Fig. 6:**
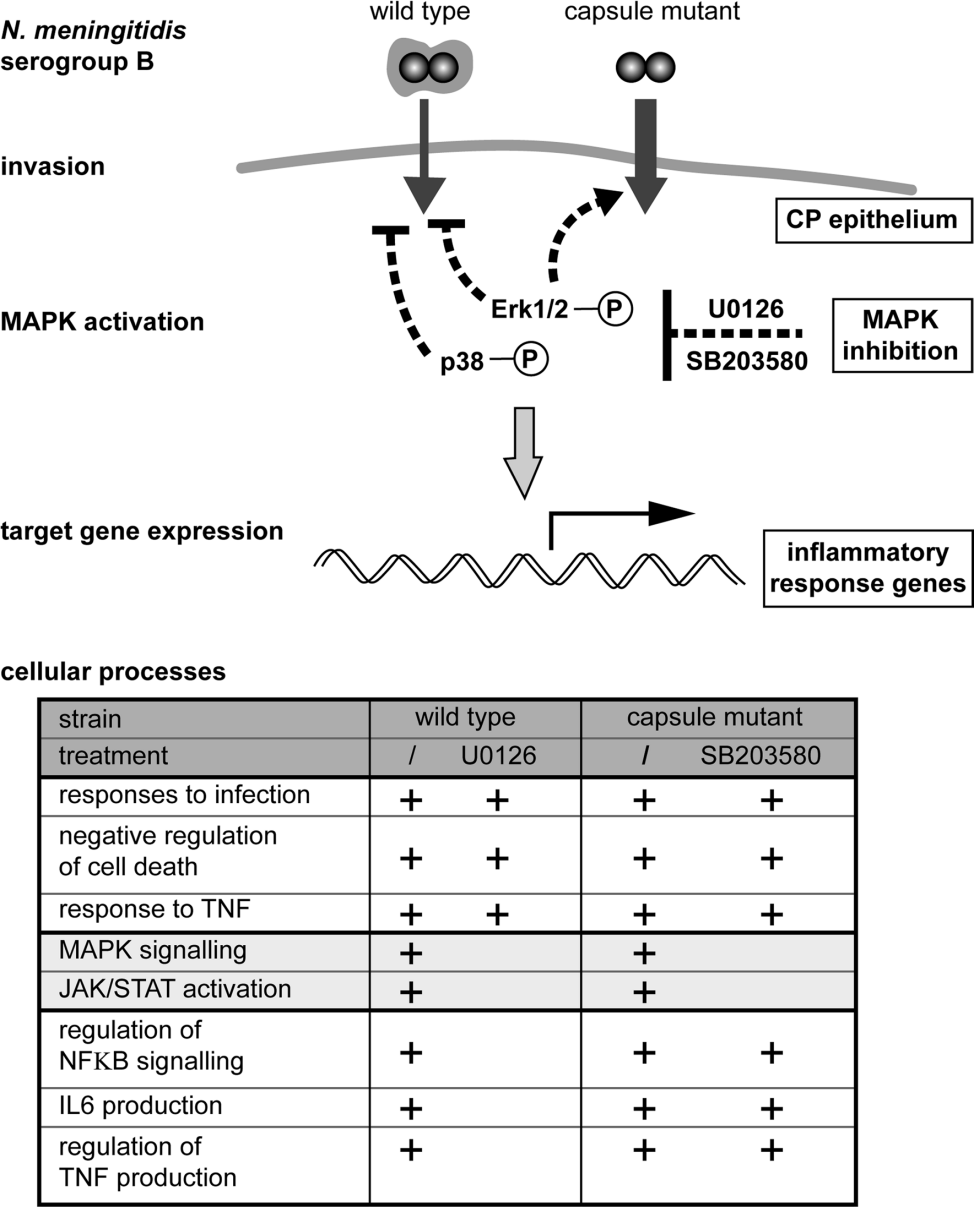
A schematic summary of the data obtained with the *Nm* serogroup B strains. Wild type and the capsule-depleted mutant of *Nm*B invade into HIBCPP cells representing the CP epithelium, with the mutant strain displaying higher invasion rates. Infection of HIBCPP cells by *Nm* leads to activation of the MAPKs Erk1/2 and p38. Inhibition of Erk1/2 and p38 with U0126 or SB203580, respectively, differentially influences the invasion by the different *Nm* strains. Whereas inhibition of Erk1/2 or p38 signalling reduces invasion by the wild type, invasion by the mutant is enhanced following inhibition of Erk1/2. Infection with wild type or capsule depleted *Nm* causes specific regulation of inflammatory response genes in the host cells, which is influenced by inhibition of Erk1/2. The impact on host cell processes cells was determined GO analysis. GO terms pointing to certain cellular processes, as production of IL-6 or regulation of TNF production, are lost specifically following Erk1/2 inhibition during infection with wild type *Nm*

A schematic summary of our data obtained with the *Nm* serogroup B strains is presented in Fig. 6. We show that invasion of HIBCPP cells by *Nm* leads to activation of MAPKs. Inhibition of MAPK signalling differentially influenced invasion by wild type and capsule-depleted bacteria, respectively. Interestingly, inhibition of Erk1/2 signalling reduced invasion by the wild type, whereas invasion by the mutant was enhanced. The impact of Erk1/2 inhibition was further reflected by differential regulation of host gene expression following infection with the wild type or then mutant. GO analysis revealed loss of GO terms related to TNF signalling and production of IL-6 specifically following Erk1/2 inhibition during infection with wild type *Nm*. This observation correlated with the reduced infection rates by the wild type in absence of Erk1/2 signalling.

Overall, intracellular pathogens, such as *Nm*, aim to establish an intracellular niche allowing for survival, replication and persistence of the pathogen. To this end, host cell signalling cascades such as the MAPK signalling cascades are manipulated and the host immune defence needs to be downregulated, while the host cell should remain healthy enough to gain nutrition [81]. During this process, bacterial effectors are crucial for targeting the host immune response at different levels with a variety of bacterial effectors mimicking host enzymes or featuring novel enzymatic functions, resulting in an overall manipulation of host cell signalling and the immune response [14, 81]. However, these complex interaction mechanisms and networks are still not completely understood. Furthermore, pathogens have been demonstrated to target different pathways simultaneously or one pathway with several effectors. Therefore, gaining further insight into the complex manipulation of host cell signalling pathways and immune response during infection will help combat infections [81]. Pharmacological inhibition of the p38 MAPK pathways in vivo and in vitro has previously been shown to effectively suppress viral replication and release, and the use of p38 inhibitors in clinical development was suggested to be trialled in COVID-19 patients, further underlining the importance of understanding the manipulation of MAPK pathways during infection for development of therapeutic approaches [82–84].

## Conclusions

Our data indicate a role of MAPK signalling during infection of *Nm* in an in vitro model of the CP epithelium. This dependency is strongly influenced by capsule expression and affects infection rates as well as the host cell response. The changes in the host cell transcriptome further point towards specific regulation of genes involved in the immune response and signalling pathways during infection. Therefore, a more detailed look into the impact of Erk1/2 signalling and its effect on the inflammatory response of the cells could give further insight on the infection mechanisms of wild type and capsule-depleted *Nm* at the BCSFB.

## Supplementary Information

**Additional file 1**. Inhibition of the Erk1/2 and p38 signalling pathways by specific inhibitors. HIBCPP cells grown in the inverted culture system and infected for 4 h with the *Nm*B strain MC58siaD^-^ (*Nm*). Untreated cells were used as controls. The activation of Erk1/2 was inhibited by adding 25 μM of the inhibitor U0126 (U) and detected using an immunoblot (A). The cells treated with PMA served as positive controls. Phosphorylation of p38 was inhibited by SB203580 (SB) at a concentration of 25 μM. The functionality of the inhibitor was examined using an immunoblot of the p38 target protein MAPKAPK-2 (B). Treatment with anisomycin (Aniso) was used as positive control.

**Additional file 2**. Summary of DEG determined by MACE Analysis of the transcriptome of HIBCPP cells during infection with *Nm*. The DEG were determined by comparing the control conditions (con, con + U0126) with the corresponding treated, infected cells. The lists shown here were filtered for a FDR p-value of < 0.05 as well as a fold change of +/- 4 (log_2_ fold change +/- 2).

**Additional file 3**. GO lists determined by the use of the software LAGO. The GO lists were created using the lists of significantly regulated DEG (see Additional file 2). A p-value cutoff of 0.01 was applied for the analysis as well as as a Bonferroni correction.

## Data Availability

The data generated during RNA-seq was deposited in the Sequence Read Archive (SRA) on the National Center for Biotechnology Information (NCBI) platform with the BioProject accession number PRJNA743158 (https://www.ncbi.nlm.nih.gov/bioproject/?term=PRJNA743158). All other data generated or analyzed during this study are included in this article and its supplementary material files. Further enquiries can be directed to the corresponding author.

## Abbreviations

ANOVA: Analysis of variance
BBB: Blood–brain barrier
BCSFB: Blood-cerebrospinal fluid barrier
CFU: Colony forming units
CNS: Central nervous system
CP: Choroid plexus
CSF: Cerebrospinal fluid
DEG: Differentially Expressed Genes
Erk: Extracellular signal-regulated kinases
FCS: Fetal calf serum
GAPDH: Glyceraldehyde 3-phosphate dehydrogenase
GO: Gene ontology
HIBCPP cells: Human epithelial choroid plexus papilloma cells
IL: Interleukin
JNK: JUN N-terminal kinases
*Lm*: *Listeria monocytogenes*
MAPK: Mitogen activated protein kinases
MAPKAP: Mitogen-activated protein kinase-activated protein kinase
MEK: MAPK/Erk kinase
MOI: Multiplicity of infection
NCBI: National Center for Biotechnology Information
*Nm*: *Neisseria meningitidis*
*Nm*B: *Nm* Serogroup B
*NmC*: *Nm* Serogroup C
OD_600_: Optical density at 600 nm
SRA: Sequence Read Archive
TEER: Transepithelial electrical resistance
UMI: Unique molecular barcodes
