# Supplementary figures and images for: Capsule-dependent impact of MAPK signalling on host cell invasion and immune response during infection of the choroid plexus epithelium by Neisseria meningitidis
Source: Fluids Barriers CNS. 2021 Dec 4;18:53. doi: 10.1186/s12987-021-00288-7 (PMC8643193; doi:10.1186/s12987-021-00288-7)

**A**

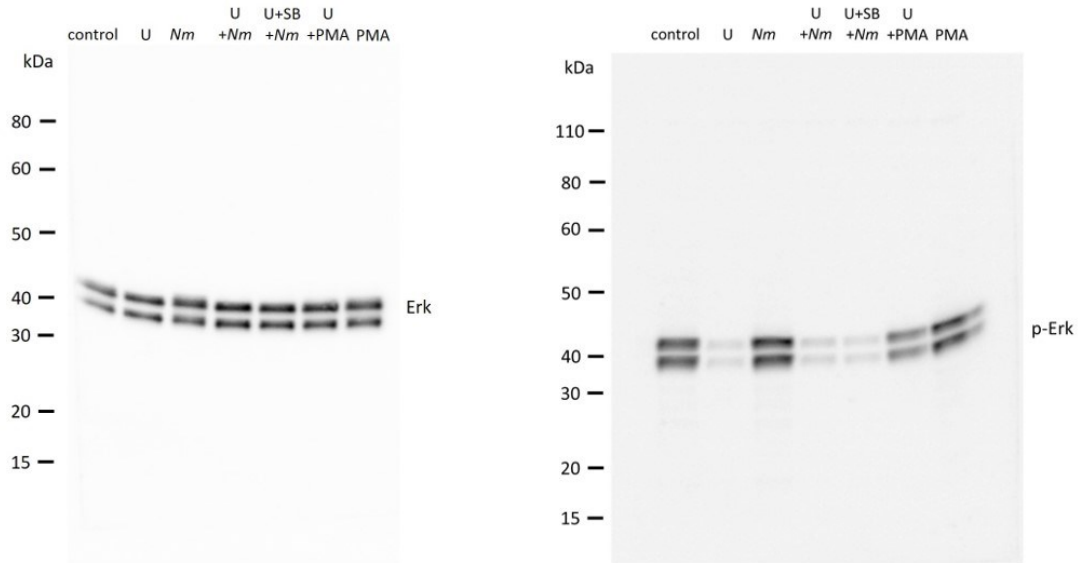

**B**

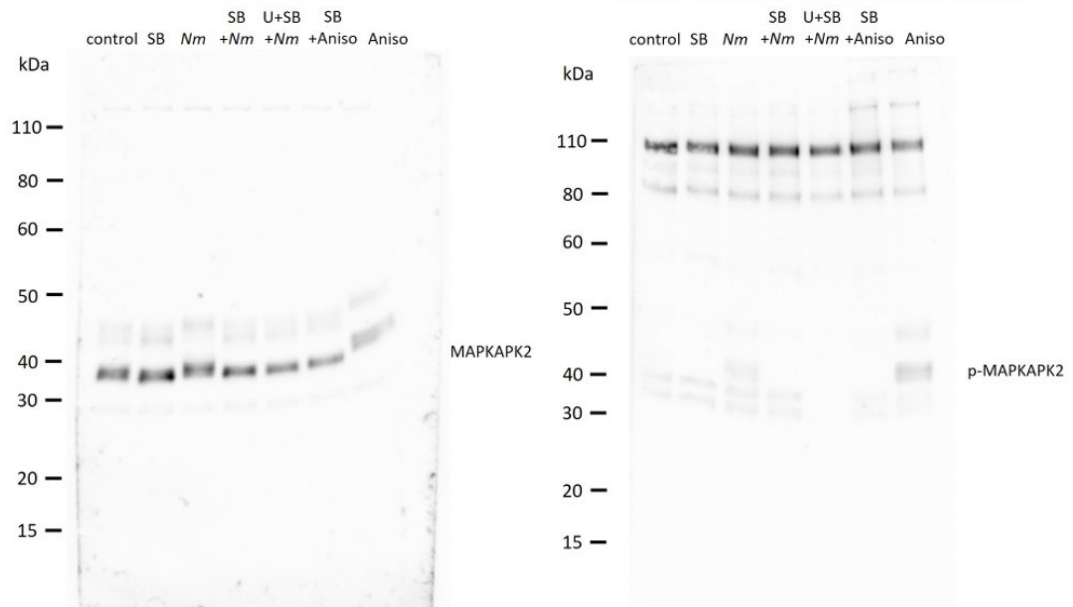

Supplement: Supplementary file 1 — Additional file 1. Inhibition of the Erk1/2 and p38 signalling pathways by specific inhibitors. HIBCPP cells grown in the inverted culture system and infected for 4 h with the NmB strain MC58siaD- (Nm). Untreated cells were used as controls. The activation of Erk1/2 was inhibited by adding 25 μM of the inhibitor U0126 (U) and detected using an immunoblot (A). The cells treated with PMA served as positive controls. Phosphorylation of p38 was inhibited by SB203580 (SB) at a concentration of 25 μM. The functionality of the inhibitor was examined using an immunoblot of the p38 target protein MAPKAPK-2 (B). Treatment with anisomycin (Aniso) was used as positive control. [file 12987_2021_288_MOESM1_ESM.pdf]
