# Supplementary material for: Capsule-dependent impact of MAPK signalling on host cell invasion and immune response during infection of the choroid plexus epithelium by Neisseria meningitidis
Source: Fluids Barriers CNS. 2021 Dec 4;18:53. doi: 10.1186/s12987-021-00288-7 (PMC8643193; doi:10.1186/s12987-021-00288-7)
Supplement: Supplementary file 2 — Additional file 2. Summary of DEG determined by MACE Analysis of the transcriptome of HIBCPP cells during infection with Nm. The DEG were determined by comparing the control conditions (con, con + U0126) with the corresponding treated, infected cells. The lists shown here were filtered for a FDR p-value of < 0.05 as well as a fold change of +/- 4 (log2 fold change +/- 2). [file 12987_2021_288_MOESM2_ESM.pdf]

| **con vs MC58** | | | |
|---|---|---|---|
| **Gene Symbol** | **Gene Name** | **Fold change** | **FDR p-value** |
| **IL1B** | interleukin 1 beta | 64,65 | 0,001022 |
| **CCL20** | C-C motif chemokine ligand 20 | 23,85 | 0,000000 |
| **IL17C** | interleukin 17C | 22,48 | 0,000000 |
| **CXCL2** | C-X-C motif chemokine ligand 2 | 20,76 | 0,000000 |
| **TNF** | tumor necrosis factor | 18,47 | 0,000000 |
| **CXCL1** | C-X-C motif chemokine ligand 1 | 15,80 | 0,000000 |
| **CXCL3** | C-X-C motif chemokine ligand 3 | 14,48 | 0,000000 |
| **CXCL8** | C-X-C motif chemokine ligand 8 | 14,03 | 0,000000 |
| **NFKBIZ** | NFKB inhibitor zeta | 13,60 | 0,000000 |
| **CSF2** | colony stimulating factor 2 | 13,01 | 0,000008 |
| **CCL2** | C-C motif chemokine ligand 2 | 12,47 | 0,000001 |
| **IL1A** | interleukin 1 alpha | 11,74 | 0,015505 |
| **ZC3H12A** | zinc finger CCCH-type containing 12A | 11,63 | 0,000000 |
| **TCIM** | transcriptional and immune response regulator | 9,73 | 0,000000 |
| **ICAM1** | intercellular adhesion molecule 1 | 9,68 | 0,000000 |
| **RND1** | Rho family GTPase 1 | 9,04 | 0,000000 |
| **CXCL6** | C-X-C motif chemokine ligand 6 | 7,92 | 0,000000 |
| **CYP24A1** | cytochrome P450 family 24 subfamily A member 1 | 7,64 | 0,000000 |
| **NFKBIA** | NFKB inhibitor alpha | 7,46 | 0,000000 |
| **TNFAIP2** | TNF alpha induced protein 2 | 7,24 | 0,000000 |
| **IL23A** | interleukin 23 subunit alpha | 6,10 | 0,000004 |
| **LIF** | LIF interleukin 6 family cytokine | 5,97 | 0,000000 |
| **PTGS2** | prostaglandin-endoperoxide synthase 2 | 5,89 | 0,000000 |
| **ANGPTL4** | angiopoietin like 4 | 5,69 | 0,000000 |
| **ZFP36** | ZFP36 ring finger protein | 5,19 | 0,000000 |
| **C6orf222** | | 4,98 | 0,021321 |
| **CX3CL1** | C-X3-C motif chemokine ligand 1 | 4,96 | 0,025366 |
| **TNFAIP3** | TNF alpha induced protein 3 | 4,94 | 0,000000 |
| **IER3** | immediate early response 3 | 4,60 | 0,000000 |
| **SLC6A14** | solute carrier family 6 member 14 | 4,59 | 0,000000 |
| **LTB** | lymphotoxin beta | 4,50 | 0,000000 |
| **CEBPD** | CCAAT enhancer binding protein delta | 4,40 | 0,000000 |
| **SOD2** | superoxide dismutase 2 | 4,30 | 0,000000 |
| **MAFF** | MAF bZIP transcription factor F | 4,21 | 0,000000 |

| **con vs MC58siaD$^-$** | | | |
|---|---|---|---|
| **Input** | **Approved name** | **Fold change** | **FDR p-value** |
| **IL1B** | interleukin 1 beta | 77,58 | 0,000399 |
| **CCL20** | C-C motif chemokine ligand 20 | 28,72 | 0,000000 |
| **IL17C** | interleukin 17C | 20,25 | 0,000000 |
| **CXCL2** | C-X-C motif chemokine ligand 2 | 19,03 | 0,000000 |
| **TNF** | tumor necrosis factor | 17,79 | 0,000000 |

| | | | |
|---|---|---|---|
| **IL6** | interleukin 6 | 16,49 | 0,016260 |
| **CXCL1** | C-X-C motif chemokine ligand 1 | 16,35 | 0,000000 |
| **CSF2** | colony stimulating factor 2 | 15,08 | 0,000001 |
| **CXCL8** | C-X-C motif chemokine ligand 8 | 14,45 | 0,000000 |
| **CXCL3** | C-X-C motif chemokine ligand 3 | 14,37 | 0,000000 |
| **CCL2** | C-C motif chemokine ligand 2 | 13,89 | 0,000000 |
| **IL1A** | interleukin 1 alpha | 11,61 | 0,015914 |
| **NFKBIZ** | NFKB inhibitor zeta | 11,15 | 0,000000 |
| **TCIM** | transcriptional and immune response regulator | 10,53 | 0,000000 |
| **ZC3H12A** | zinc finger CCCH-type containing 12A | 10,14 | 0,000000 |
| **CXCL6** | C-X-C motif chemokine ligand 6 | 9,75 | 0,000000 |
| **ICAM1** | intercellular adhesion molecule 1 | 9,46 | 0,000000 |
| **RND1** | Rho family GTPase 1 | 9,08 | 0,000000 |
| **CYP24A1** | cytochrome P450 family 24 subfamily A member 1 | 8,70 | 0,000000 |
| **IL23A** | interleukin 23 subunit alpha | 8,24 | 0,000000 |
| **TNFAIP2** | TNF alpha induced protein 2 | 7,81 | 0,000000 |
| **NFKBIA** | NFKB inhibitor alpha | 7,34 | 0,000000 |
| **SPIB** | Spi-B transcription factor | 6,49 | 0,022577 |
| **RASD1** | ras related dexamethasone induced 1 | 6,34 | 0,039433 |
| **IGFBP1** | insulin like growth factor binding protein 1 | 6,27 | 0,036090 |
| **LTB** | lymphotoxin beta | 5,92 | 0,000000 |
| **PTGS2** | prostaglandin-endoperoxide synthase 2 | 5,68 | 0,000000 |
| **ADORA2A** | adenosine A2a receptor | 5,44 | 0,000219 |
| **ANGPTL4** | angiopoietin like 4 | 5,34 | 0,000000 |
| **SOD2** | superoxide dismutase 2 | 5,21 | 0,000000 |
| **LIF** | LIF interleukin 6 family cytokine | 5,01 | 0,000000 |
| **SLC6A14** | solute carrier family 6 member 14 | 4,97 | 0,000000 |
| **CSF1** | colony stimulating factor 1 | 4,77 | 0,003530 |
| **PI3** | peptidase inhibitor 3 | 4,73 | 0,014113 |
| **SERPINA3** | serpin family A member 3 | 4,71 | 0,000000 |
| **IER3** | immediate early response 3 | 4,60 | 0,000000 |
| **CX3CL1** | C-X3-C motif chemokine ligand 1 | 4,59 | 0,044719 |
| **ZFP36** | ZFP36 ring finger protein | 4,48 | 0,000003 |
| **CEBPD** | CCAAT enhancer binding protein delta | 4,47 | 0,000000 |
| **TNFAIP3** | TNF alpha induced protein 3 | 4,45 | 0,000000 |
| **TNFRSF6B** | TNF receptor superfamily member 6b | 4,44 | 0,000000 |
| **HILPDA** | hypoxia inducible lipid droplet associated | 4,27 | 0,000000 |
| **MAFF** | MAF bZIP transcription factor F | 4,13 | 0,000000 |

| **con vs con + U0126** | | | |
|---|---|---|---|
| **Input** | **Approved name** | **Fold change** | **FDR p-value** |
| **DUSP6** | dual specificity phosphatase 6 | 9,73 | 0,000000 |
| **CLDN2** | claudin 2 | 9,47 | 0,000275 |
| **ANKRD37** | ankyrin repeat domain 37 | 7,72 | 0,000000 |
| **EGR1** | early growth response 1 | 6,78 | 0,044671 |

| | | | |
|---|---|---|---|
| **ETV5** | ETS variant transcription factor 5 | 6,52 | 0,000004 |
| **ANGPTL4** | angiopoietin like 4 | 4,17 | 0,000005 |
| **CYP1B1** | cytochrome P450 family 1 subfamily B member 1 | -4,20 | 0,000000 |
| **LOC105374986** | | -5,01 | 0,000105 |
| **TNFSF12** | TNF superfamily member 12 | -5,03 | 0,006733 |
| **VASN** | vasorin | -5,41 | 0,000403 |
| **NUPR1** | nuclear protein 1, transcriptional regulator | -5,76 | 0,000908 |
| **SECTM1** | secreted and transmembrane 1 | -6,30 | 0,000001 |
| **AHRR** | aryl-hydrocarbon receptor repressor | -7,48 | 0,000000 |
| **BMF** | Bcl2 modifying factor | -13,19 | 0,000000 |

| **Con + U0126 vs MC58 + U0126** | | | |
|---|---|---|---|
| **Input** | **Approved name** | **Fold change** | **FDR p-value** |
| **IL17C** | interleukin 17C | 79,66 | 0,000425 |
| **CCL20** | C-C motif chemokine ligand 20 | 20,68 | 0,000000 |
| **CXCL2** | C-X-C motif chemokine ligand 2 | 20,30 | 0,000000 |
| **TNF** | tumor necrosis factor | 18,21 | 0,000000 |
| **CXCL1** | C-X-C motif chemokine ligand 1 | 16,79 | 0,000000 |
| **CXCL8** | C-X-C motif chemokine ligand 8 | 16,17 | 0,000000 |
| **CXCL3** | C-X-C motif chemokine ligand 3 | 15,66 | 0,000000 |
| **ZC3H12A** | zinc finger CCCH-type containing 12A | 12,04 | 0,000000 |
| **CCL2** | C-C motif chemokine ligand 2 | 11,80 | 0,000000 |
| **ANKRD37** | ankyrin repeat domain 37 | 10,05 | 0,000000 |
| **NFKBIZ** | NFKB inhibitor zeta | 8,18 | 0,000000 |
| **ICAM1** | intercellular adhesion molecule 1 | 7,98 | 0,000000 |
| **CXCL6** | C-X-C motif chemokine ligand 6 | 7,67 | 0,000000 |
| **ANGPTL4** | angiopoietin like 4 | 7,59 | 0,000000 |
| **NFKBIA** | NFKB inhibitor alpha | 7,33 | 0,000000 |
| **RND1** | Rho family GTPase 1 | 7,02 | 0,000000 |
| **ADORA2A** | adenosine A2a receptor | 6,91 | 0,014927 |
| **TCIM** | transcriptional and immune response regulator | 6,77 | 0,000000 |
| **ADM** | adrenomedullin | 6,36 | 0,000000 |
| **PFKFB4** | 6-phosphofructo-2-kinase/fructose-2,6-biphosphatase 4 | 6,34 | 0,000000 |
| **CX3CL1** | C-X3-C motif chemokine ligand 1 | 6,12 | 0,003608 |
| **C6orf222** | | 5,77 | 0,006118 |
| **MIR210HG** | MIR210 host gene | 5,63 | 0,000105 |
| **TNFAIP2** | TNF alpha induced protein 2 | 5,59 | 0,000000 |
| **IL4I1** | interleukin 4 induced 1 | 5,48 | 0,000001 |
| **CXCL10** | C-X-C motif chemokine ligand 10 | 5,40 | 0,011115 |
| **TNFAIP3** | TNF alpha induced protein 3 | 5,03 | 0,000000 |
| **FBXL8** | F-box and leucine rich repeat protein 8 | 4,75 | 0,041838 |
| **BHLHE40** | basic helix-loop-helix family member e40 | 4,36 | 0,000000 |
| **SOD2** | superoxide dismutase 2 | 4,09 | 0,000000 |
| **CEBPD** | CCAAT enhancer binding protein delta | 4,01 | 0,000000 |

| Con + U0126 vs MC58siaD$^-$ + U0126 | | | |
|---|---|---|---|
| **Input** | **Approved name** | **Fold change** | **FDR p-value** |
| **IL17C** | interleukin 17C | 190,68 | 0,000002 |
| **IL1B** | interleukin 1 beta | 56,80 | 0,002956 |
| **CCL20** | C-C motif chemokine ligand 20 | 37,32 | 0,000000 |
| **IL1A** | interleukin 1 alpha | 28,19 | 0,003607 |
| **CXCL8** | C-X-C motif chemokine ligand 8 | 26,67 | 0,000000 |
| **CXCL2** | C-X-C motif chemokine ligand 2 | 26,60 | 0,000000 |
| **TNF** | tumor necrosis factor | 26,58 | 0,000000 |
| **CXCL1** | C-X-C motif chemokine ligand 1 | 25,62 | 0,000000 |
| **CCL2** | C-C motif chemokine ligand 2 | 22,91 | 0,000000 |
| **CXCL3** | C-X-C motif chemokine ligand 3 | 21,85 | 0,000000 |
| **CSF2** | colony stimulating factor 2 | 15,09 | 0,000273 |
| **ZC3H12A** | zinc finger CCCH-type containing 12A | 14,27 | 0,000000 |
| **CXCL6** | C-X-C motif chemokine ligand 6 | 13,31 | 0,000000 |
| **ANKRD37** | ankyrin repeat domain 37 | 12,78 | 0,000000 |
| **ADORA2A** | adenosine A2a receptor | 11,67 | 0,000061 |
| **ICAM1** | intercellular adhesion molecule 1 | 11,60 | 0,000000 |
| **RND1** | Rho family GTPase 1 | 11,45 | 0,000000 |
| **ANGPTL4** | angiopoietin like 4 | 10,50 | 0,000000 |
| **TCIM** | transcriptional and immune response regulator | 10,40 | 0,000000 |
| **IL4I1** | interleukin 4 induced 1 | 9,49 | 0,000000 |
| **NFKBIA** | NFKB inhibitor alpha | 9,29 | 0,000000 |
| **CXCL10** | C-X-C motif chemokine ligand 10 | 8,81 | 0,000033 |
| **NFKBIZ** | NFKB inhibitor zeta | 8,80 | 0,000000 |
| **CX3CL1** | C-X3-C motif chemokine ligand 1 | 8,78 | 0,000034 |
| **NOS2** | nitric oxide synthase 2 | 8,64 | 0,023055 |
| **IL6** | interleukin 6 | 8,56 | 0,016570 |
| **TNFAIP2** | TNF alpha induced protein 2 | 8,00 | 0,000000 |
| **SAA2** | serum amyloid A2 | 7,67 | 0,015618 |
| **C6orf222** | BCL2 interacting protein 5 | 7,67 | 0,000176 |
| **GFPT2** | glutamine-fructose-6-phosphate transaminase 2 | 7,60 | 0,009101 |
| **PFKFB4** | 6-phosphofructo-2-kinase/fructose-2,6-biphosphatase 4 | 7,43 | 0,000000 |
| **ADM** | adrenomedullin | 7,28 | 0,000000 |
| **IL23A** | interleukin 23 subunit alpha | 7,28 | 0,000004 |
| **TNFRSF6B** | TNF receptor superfamily member 6b | 7,26 | 0,000000 |
| **SOD2** | superoxide dismutase 2 | 7,17 | 0,000000 |
| **MIR210HG** | MIR210 host gene | 6,90 | 0,000003 |
| **CHAC1** | ChaC glutathione specific gamma-glutamylcyclotransferase 1 | 6,48 | 0,000065 |
| **SERPINA3** | serpin family A member 3 | 6,36 | 0,000000 |
| **SLC6A14** | solute carrier family 6 member 14 | 6,04 | 0,000000 |
| **ANKK1** | ankyrin repeat and kinase domain containing 1 | 5,99 | 0,001924 |

| | | | |
|---|---|---|---|
| **LTB** | lymphotoxin beta | 5,95 | 0,000000 |
| **TNFAIP3** | TNF alpha induced protein 3 | 5,48 | 0,000000 |
| **G0S2** | G0/G1 switch 2 | 5,46 | 0,000000 |
| **ZC3H12C** | zinc finger CCCH-type containing 12C | 5,44 | 0,039084 |
| **LOC101929444** | | 5,29 | 0,000252 |
| **HILPDA** | hypoxia inducible lipid droplet associated | 4,99 | 0,000000 |
| **CEBPD** | CCAAT enhancer binding protein delta | 4,97 | 0,000000 |
| **TNFRSF11B** | TNF receptor superfamily member 11b | 4,90 | 0,000006 |
| **EGLN3** | egl-9 family hypoxia inducible factor 3 | 4,70 | 0,000000 |
| **CYP24A1** | cytochrome P450 family 24 subfamily A member 1 | 4,63 | 0,000000 |
| **PI3** | peptidase inhibitor 3 | 4,62 | 0,021402 |
| **PFKFB3** | 6-phosphofructo-2-kinase/fructose-2,6-biphosphatase 3 | 4,59 | 0,000000 |
| **RCAN1** | regulator of calcineurin 1 | 4,54 | 0,000000 |
| **PTGS2** | prostaglandin-endoperoxide synthase 2 | 4,50 | 0,000002 |
| **BHLHE40** | basic helix-loop-helix family member e40 | 4,38 | 0,000000 |
| **PLAU** | plasminogen activator, urokinase | 4,02 | 0,000000 |
